# Isogenic models of hypertrophic cardiomyopathy unveil differential phenotypes and mechanism-driven therapeutics

**DOI:** 10.1016/j.yjmcc.2020.06.003

**Published:** 2020-08

**Authors:** Jamie R. Bhagwan, Diogo Mosqueira, Karolina Chairez-Cantu, Ingra Mannhardt, Sara E. Bodbin, Mine Bakar, James G.W. Smith, Chris Denning

**Affiliations:** a Division of Cancer & Stem Cells, Biodiscovery Institute, University of Nottingham, NG7 2RD, UK; b Department of Experimental Pharmacology and Toxicology, Cardiovascular Research Center, University Medical Center Hamburg-Eppendorf, and DZHK (German Center for Cardiovascular Research), partner site Hamburg/Kiel/Lübeck, Hamburg, Germany; c Faculty of Medicine and Health Sciences, Norwich Medical School, University of East Anglia,NR4 7UQ, UK

**Keywords:** Hypertrophic cardiomyopathy, Disease modeling, Isogenic human pluripotent stem cell-derived cardiomyocytes, Mechanistic insight, Tailored therapeutics

## Abstract

**Background:**

Hypertrophic cardiomyopathy (HCM) is a prevalent and complex cardiovascular condition. Despite being strongly associated with genetic alterations, wide variation of disease penetrance, expressivity and hallmarks of progression complicate treatment. We aimed to characterize different human isogenic cellular models of HCM bearing patient-relevant mutations to clarify genetic causation and disease mechanisms, hence facilitating the development of effective therapeutics.

**Methods:**

We directly compared the p.β-MHC-R453C and p.ACTC1-E99K HCM-associated mutations in human induced pluripotent stem cell-derived cardiomyocytes (hiPSC-CMs) and their healthy isogenic counterparts, generated using CRISPR/Cas9 genome editing technology. By harnessing several state-of-the-art HCM phenotyping techniques, these mutations were investigated to identify similarities and differences in disease progression and hypertrophic signaling pathways, towards establishing potential targets for pharmacological treatment. CRISPR/Cas9 knock-in of the genetically-encoded calcium indicator R-GECO1.0 to the *AAVS1* locus into these disease models resulted in calcium reporter lines.

**Results:**

Confocal line scan analysis identified calcium transient arrhythmias and intracellular calcium overload in both models. The use of optogenetics and 2D/3D contractility assays revealed opposing phenotypes in the two mutations. Gene expression analysis highlighted upregulation of *CALM1, CASQ2* and *CAMK2D,* and downregulation of *IRF8* in p.β-MHC-R453C mutants, whereas the opposite changes were detected in p.ACTC1-E99K mutants. Contrasting profiles of nuclear translocation of NFATc1 and MEF2 between the two HCM models suggest differential hypertrophic signaling pathway activation. Calcium transient abnormalities were rescued with combination of dantrolene and ranolazine, whilst mavacamten reduced the hyper-contractile phenotype of p.ACTC1-E99K hiPSC-CMs.

**Conclusions:**

Our data show that hypercontractility and molecular signaling within HCM are not uniform between different gene mutations, suggesting that a ‘one-size fits all’ treatment underestimates the complexity of the disease. Understanding where the similarities (arrhythmogenesis, bioenergetics) and differences (contractility, molecular profile) lie will allow development of therapeutics that are directed towards common mechanisms or tailored to each disease variant, hence providing effective patient-specific therapy.

## Highlights

• Isogenic models of hiPSC-CMs bearing sarcomeric mutations address HCM complexity.
• Calcium reporter lines and optogenetics enabled refined phenotypic analyses of HCM.
• Different mutations showed phenotypic and mechanistic similarities and differences.
• Arrhythmias in both models were rescued by combining ranolazine and dantrolene.
• Variable phenotypes support mechanism-driven development of effective therapeutics.

## Graphical abstract

**Figure:**
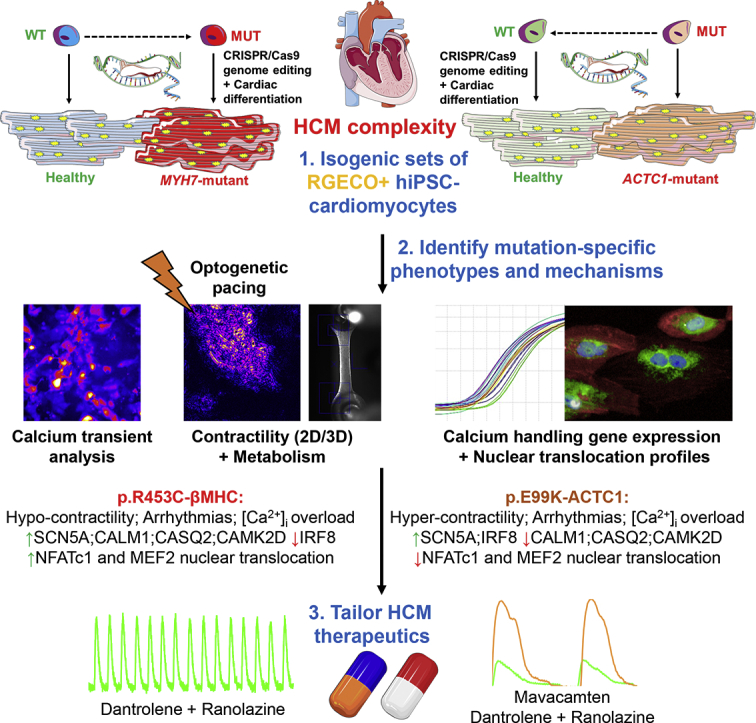

## 1 Introduction

Hypertrophic cardiomyopathy (HCM) is a prevalent cardiovascular disease which is the leading cause of sudden cardiac death in young adults, including athletes [1,2]. Patients with HCM have a diverse clinical presentation, although thickening of the left ventricle detected by echocardiogram is commonly reported [3]. Other clinical manifestations include cardiomyocyte hypertrophy, myofibrillar disarray and left ventricular outflow tract (LVOT) obstruction [4].

Over 1400 mutations across over 25 genes have been associated with HCM progression, with the majority affecting sarcomeric proteins, which regulate cardiomyocyte contraction [5]. Of the sarcomeric genes involved, 20–50% of genotyped cases implicate *MYH7*, encoding for the beta myosin heavy chain (β-MHC) thick filament. A lower incidence (1–5%) involve *ACTC1*, encoding the alpha cardiac actin thin filament [6]. However, approximately half of HCM patients do not exhibit mutations in known sarcomeric genes [7], and while genetic lesions occur at 1:200 incidence, only 1:500 individuals develop HCM phenotypes [8]. Thus, with such an array of potentially causative mutations, HCM has variable penetrance, expressivity and severity [9], which greatly complicates treatment.

Due to this phenotypic variability, various animal models, explanted heart biopsies and their derivatives, and in vitro cellular models have been developed to gain a deeper understanding of this complex disease. However, the investigation of different disease models has often led to conflicting results. The study of animals is hampered by species-differences in pathophysiology, whilst processed tissue typically involves demanding logistics and/or tendency towards technical artefacts [10]. Some models have linked sarcomeric mutations to higher myofilament calcium sensitivity [11,12] and arrhythmias [13,14]. Others have shown altered contractility but this varies from hypo- [[15], [16], [17]], to hyper-contractility [[18], [19], [20]]. It is now clear that HCM phenotypes are mutation-specific, and their detection and validation depends on the disease model under study and the existence of *bonafide* (isogenic) genetic controls [21], which were, until recent advances in gene-editing technology, challenging to obtain.

Given this variability, we sought to directly compare two isogenic sets of HCM in physiologically-relevant human induced pluripotent stem cell-cardiomyocyte (hiPSC-CM) models. We have previously demonstrated the CRISPR/Cas9 generation of an isogenic trio of wild-type, heterozygote and homozygote hiPSCs harbouring the g.*MYH7*^C9123T^ mutation, corresponding to the pathogenic protein change p.β-MHC-R453C [15]. In addition, we developed an isogenic duo of hiPSCs harbouring the c.*ACTC1*^G301A^ mutation, also known as p.ACTC1-E99K [19]. These hiPSC-CMs models were focused on the validation of key HCM hallmarks (hypertrophy and activation of brain natriuretic peptide (BNP) pathway, sarcomeric disarray and calcium sensitivity) and provided clarification of the genetic causation of this disease.

Here, we extended these studies by performing parallel comparisons of the cellular and molecular features of these two in vitro hiPSC-CM models of HCM, to identify mutation-specific effects that could better inform clinicians on the most efficient therapeutic option. By utilising state-of-the-art gene-edited isogenic controls and phenotyping technology [22], the molecular characterization of refined cellular models of HCM enabled deeper understanding of mechanistic complexity and addressed the need for efficient therapy.

## 2 Materials and methods

Detailed methods are provided in the online Supplementary Materials. All patient skin biopsies were donated via informed consent under the approval of Research Ethics Committee - number 09/H0408/74. All data is presented as mean with standard deviation (SD) or box and whiskers plot, with the number of biological replicates indicated in the respective figure legend, performed in technical triplicates unless otherwise stated. Statistical analysis was performed using GraphPad software (v8.2). *ACTC1*^MUT/WT^ was directly compared to *ACTC1*^WT/WT^ hiPSC-CMs using unpaired Student's *t*-test. A one-way ANOVA with Newman-Keuls multiple comparison test was used for *MYH7* isogenic trio data analysis, unless otherwise stated. Drug treatments were compared to vehicle controls using unpaired Student's t-test. Significance tests were based on *p*-values as follows: * *p* < .05; **p < .01; ***p < .001; ****p < .0001.

## 3 Results

### 3.1 Revisiting isogenic sets of *ACTC1*-mutant and *MYH7*-mutant hiPSCs generated by CRISPR/Cas9 editing

In order to compare the effects of different sarcomeric mutations in HCM, we have utilized previously generated isogenic hiPSC-CM models, whereby CRISPR/Cas9 was used to either introduce the g.*MYH7*^C9123T^mutation in healthy hiPSCs or correct the c.*ACTC1*^G301A^variant in patient lines. In each case, a gRNA/Cas9-nickase/CRISPR strategy was used to introduce a drug selection cassette concurrently with the polymorphism of interest in the juxtaposed arm of homology. After confirmation of targeting, recombinase-mediated cassette excision was employed to create the isogenic sets [23], the fidelity of which were confirmed by PCR genotyping and sequencing. These five cell lines comprised a trio of wild-type (*MYH7*^WT/WT^), heterozygous (*MYH7*^WT/MUT^) and homozygous (*MYH7*^MUT/MUT^) mutants for the p.β-MHC-R453C change (**Fig. S1A-C**) derived from a health line named REBL-PAT, and a duo of wild-type (*ACTC1*^WT/WT^) and heterozygous (*ACTC1*^WT/MUT^) for the p.ACTC1-E99K change (**Fig. S1D—F**) originated from a heterozygous patient (E99K1) [19]. These isogenic sets validated the hypertrophic phenotype, as mutant hiPSC-cardiomyocytes displayed the main hallmarks of HCM such as increase in cell volume, activation of the hypertrophic BNP pathway and sarcomeric disarray [15,19].

### 3.2 Generation of calcium reporter lines by CRISPR/Cas9 targeting of the *AAVS1* safe harbor locus

**Fig. 1:**
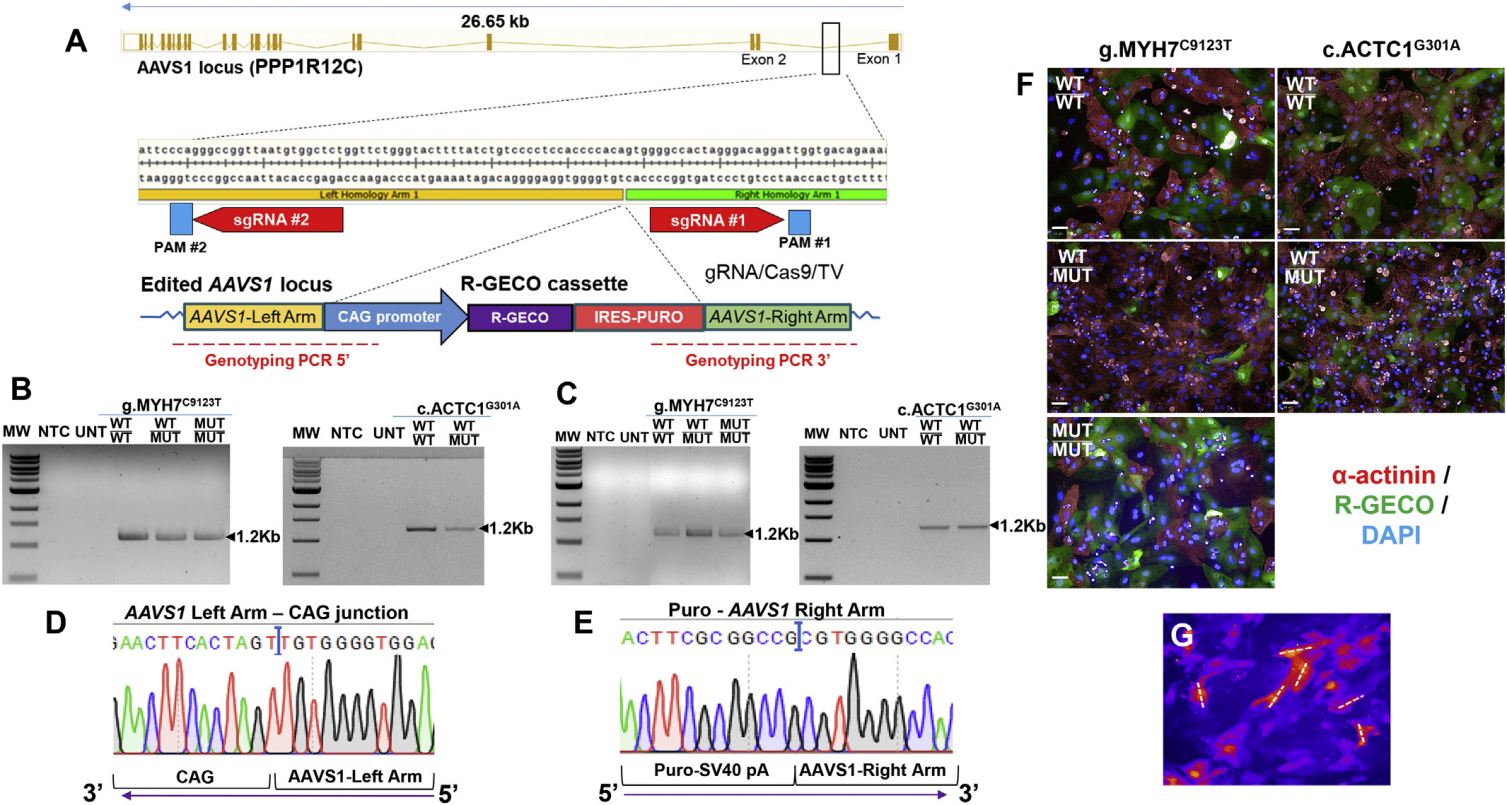
Generation of GECI lines by CRISPR/Cas9 targeting of the *AAVS1* safe harbor locus. **A)** CRISPR/Cas9 nickase strategy was used to knock-in a CAG-driven R-GECO cassette in an intron of the *AAVS1* locus in hiPSC-CM lines. **B,C)** PCR-genotyping confirms insertion of the R-GECO cassette in the AAVS1 locus of g.MYH7^C9123T^ and c.ACTC1^G301A^ HCM lines and their isogenic controls (correct product sizes indicated). **D,E)** Correct gene-editing was verified by Sanger sequencing the junctions between left and right arms of homology with the respective cassette ends. **F)** Representative fluorescence micrographs illustrating expression of R-GECO (green) in all the generated hiPSC-CM (red) lines (scale bar = 50 μm). **G)** Confocal line scans were used to perform calcium imaging in hiPSC-CMs, by measuring the fluorescence intensity in the delineated region, in a single cell. TV, Targeting Vector; MW, molecular weight; NTC, non-template control; UNT, untargeted; WT, wild-type; MUT, mutant. (For interpretation of the references to colour in this figure legend, the reader is referred to the web version of this article.)

In order to assess the effect of each mutation on calcium transients, CRISPR/Cas9 was used to introduce the genetically-encoded calcium indicator (GECI) R-GECO1.0, into the genome of the five hiPSC lines. The safe harbor locus *AAVS1* was targeted, interrupting the first intron of *PPP1R12C* (Fig. 1A), which has been reported to be devoid of any adverse cellular effects [24]. A dual gRNA/Cas9-nickase/CRISPR approach was used to knock-in a cassette containing R-GECO1.0 driven by the CAG promoter, followed by a puromycin resistance gene. This cassette was flanked on each side with 1 kb of homology to the *AAVS1* locus.

Successful targeting of the *AAVS1* locus was confirmed by PCR genotyping and sequencing in each of the five genetic backgrounds (Fig. 1B-E). These hiPSC lines were successfully differentiated to hiPSC-CMs, and expression of R-GECO1.0 was verified by immunocytochemistry for α-actinin and RFP, with the latter antibody cross reacting with R-GECO1.0 (Fig. 1F). Despite the initially observed heterogeneity in the RGECO1.0 expression between hiPSC-CM clones, due to transcriptional silencing upon differentiation (as recently reported [25]), we have minimized interline differences by choosing clones with comparable RGECO expression levels for phenotypic studies. Moreover, the use of a GECI ensured higher longevity of the calcium signal without increasing the background fluorescence over time (**Fig. S2A,B**), and no cytotoxicity or signal diffusion (**Fig. S2C-E**) relative to the commonly used Fluo-4 AM calcium dye. The reporter lines displayed comparable cardiomyocyte differentiation efficiencies at >90% (**Fig. S3A,B**) and enabled calcium imaging by confocal line scans of single cardiomyocytes (Figs. 1G**, S3C**), as an alternative to multicellular approaches based on the evaluation of rotor formation in cardiomyocyte monolayers labelled with calcium-dyes [26].

### 3.3 Confocal laser line scan analysis highlights calcium transient abnormalities in both *ACTC1-* and *MYH7-*mutant hiPSC-CMs

**Fig. 2:**
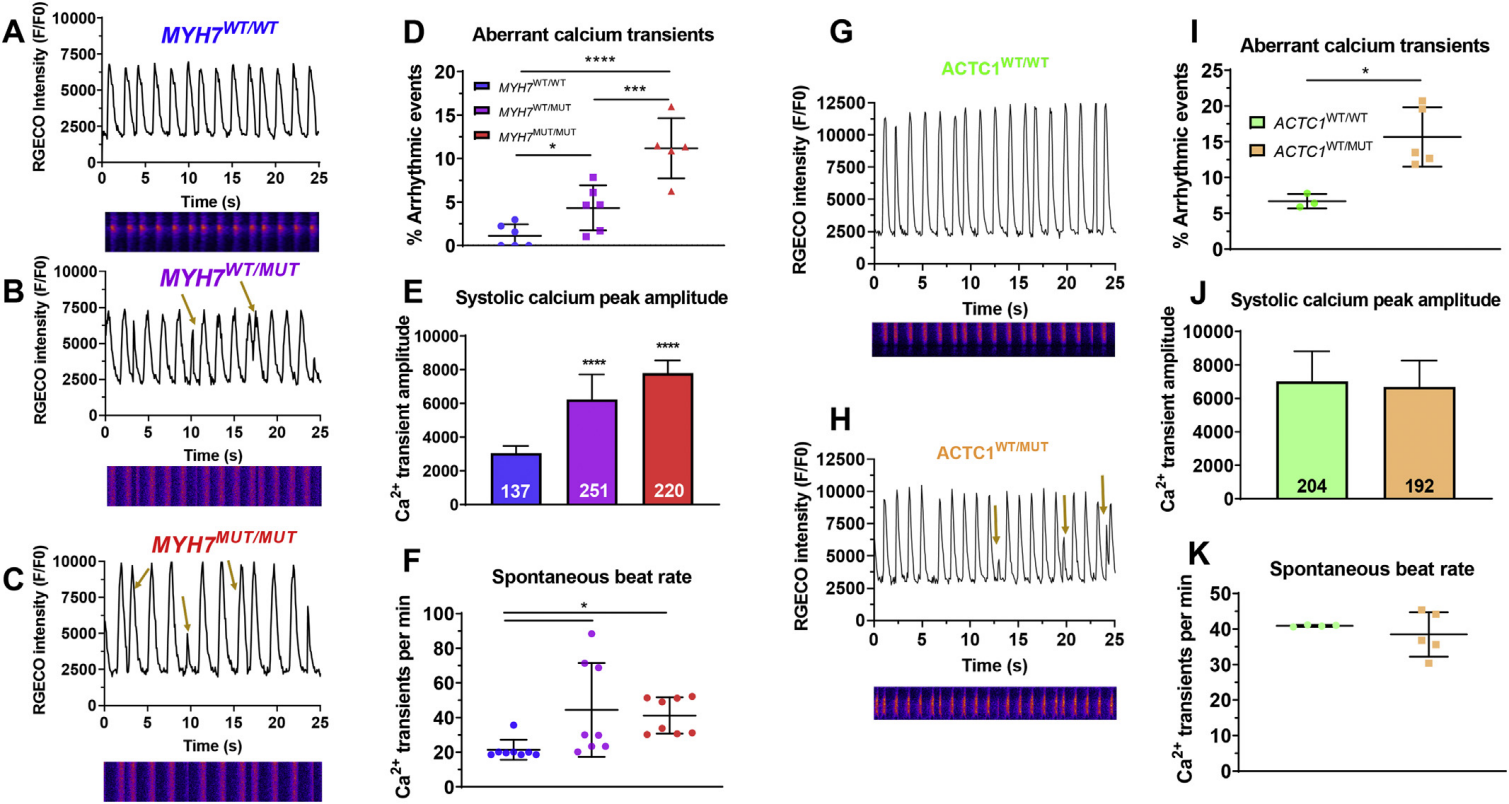
Calcium transient analysis underlying arrhythmias in HCM hiPSC-CM lines. **A-C)** Confocal line scanning of g.MYH7^C9123T^ isogenic trio highlighted abnormal calcium transient events (golden arrows). **D)** Mean frequency of arrhythmic events increased from 1.13% in *MYH7*^WT/WT^ hiPSC-CMs to 4.33% in heterozygous *MYH7*^C9123T^ mutants and 11.20% in the isogenic homozygous line. *MYH7*-mutant hiPSC-CMs exhibited: **E)** higher amplitude of calcium signal relative to isogenic WT control, suggesting an elevated systolic calcium peak (~2-fold higher in the heterozygous line, ~2.6-fold-higher in the homozygous line), and **F)** faster beat rate (average 21 bpm for WT, 44 bpm for heterozygous mutant and 41 bpm for homozygous mutant cardiomyocytes). **G-I)** The c.ACTC1^G301A^ mutant hiPSC-CMs also displayed aberrant calcium transients (15.7% vs 6.7% in isogenic healthy control), with **J)** no significant changes in Ca^2+^ signal amplitude and **K)** beat rate, relative to isogenic WT control. Data: mean ± SD, number of technical replicates indicated in respective bars in E and J, one-way ANOVA + Newman-Keuls correction for multiple comparisons in *MYH7* isogenic trio (D-F: *N* = 6–8 independent biological replicates) and unpaired *t*-test between *ACTC1* lines (I-K: *N* = 3–4 independent biological replicates), *p < .05, ***p < .001, ****p < .0001.

Calcium transient abnormalities have been identified as a key phenotype of HCM [13], so we sought to assess this in both *ACTC1* and *MYH7-*mutant hiPSC-CMs utilising the expression of R-GECO1.0. For this, hiPSCs were differentiated in a monolayer protocol [15], re-plated as single cells at day 15, and calcium transient analysis was performed at day 30, through rapid laser line confocal microscopy at 1.8 mM Ca^2+^. Aberrant calcium transient events manifested as double peaks and/or partial peaks, and could be quantified using pClamp software. Frequency of aberrant events increased in accordance with mutation load by 4- to 10-fold for heterozygous and homozygous *MYH7*, respectively (Fig. 2A-D), and 2.3-fold for heterozygous *ACTC1* (Fig. 2G-I).

We hypothesised that these abnormal events indicated arrhythmias and occurred as a result of high cytosolic calcium levels. Therefore, the amplitude of the fluorescence signal-to-noise ratio (*F/F0*) was used as approximation to gain an insight into the levels of intracellular calcium. In both *MYH7*^WT/MUT^ and *MYH7*^MUT/MUT^ hiPSC-CMs, systolic calcium peak amplitude was significantly greater than the *MYH7*^WT/WT^ isogenic control (Fig. 2E). However, this was not recapitulated in *ACTC1*^WT/MUT^ hiPSC-CMs (Fig. 2J). In addition, we have measured the baseline RGECO fluorescence intensity (F0) as an approximation of the diastolic calcium levels in hiPSC-CMs. These showed that HCM lines displayed higher diastolic calcium reserves relative to their isogenic healthy lines, in both mutations (**Fig. S3D,E**). Moreover, we assessed the sarcoplasmic reticulum (SR) Ca^2+^storage capacity by analysing calcium transient activity upon treatment with 10 mM caffeine, which induces Ca^2+^release into the cytoplasm. This revealed similar SR Ca^2+^release (RGECO ∆F/F0 peak intensity ratio) between HCM-mutant hiPSC-CMs and their healthy isogenic controls (**Fig. S3F-H**).

Other differences were also evident between *MYH7* and *ACTC1* mutants. Namely, while *MYH7*-mutant hiPSC-CMs displayed increased spontaneous beat rate relative to isogenic WT control (Fig. 2F), the *ACTC1* mutation did not cause significant changes in beat rate (Fig. 2K). Taken together, these data showed that aberrant calcium transient events were a common feature between the gene mutations, but there were key differences in the response in systolic calcium peak amplitude and spontaneous beat rate for *MYH7* relative to *ACTC1* mutants.

### 3.4 Contractility analysis utilising 2D optogenetic pacing and 3D engineered heart tissue technology reveals opposing phenotypes between HCM models

To provide a functional characterization of the two isogenic HCM model lines, contractility was assessed in both 2D and 3D formats. For 2D contractility analysis, hiPSC-CMs were first transiently transfected with a vector containing a CAG-ChR2-EYFP-T2A-BSD cassette, at an average efficiency across all five HCM lines of ~38% (**Fig. S4**). Expression of channelrhodopsin-2 (ChR2) allowed hiPSC-CMs to be optically paced at 1 Hz with LED light, obviating the need for invasive electrode pacing.

**Fig. 3:**
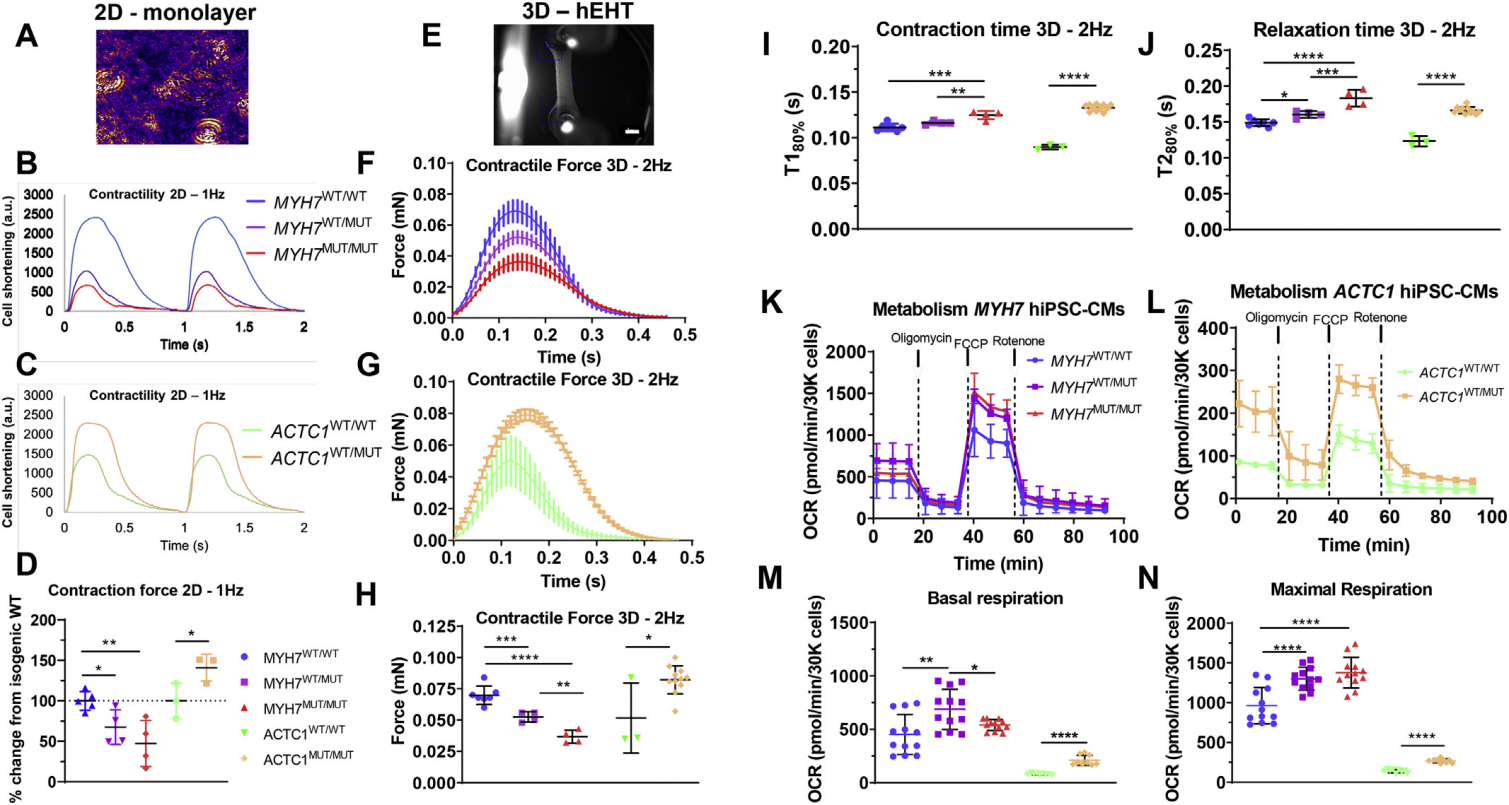
Functional phenotyping of contractility and metabolism of HCM hiPSC-CMs. **A)** Micrograph showing pixel displacement of monolayer of hiPSC-CMs, which enables estimation of contractility in 2D by CellOPTIQ® technology. **B,C)** Representative traces of optically-paced (1 Hz) ChR2^+^ cardiomyocytes highlight a hypo-contractile phenotype in *g.MYH7*^*C9123T*^ mutants and hyper-contractility in *c.ACTC1*^*G301A*^ variants. **D)** Peak amplitude quantification revealed that *MYH7*^WT/MUT^ and *MYH7*^MUT/MUT^ hiPSC-CMs displayed a reduction in contractility relative to the isogenic WT control by ~32% and ~ 53%, respectively (*N* = 5 independent biological replicates), whereas *ACTC1*-mutant hiPSC-CMs exhibited ~41% higher amplitude relative to its isogenic control (*N* = 3 independent biological replicates). **E)** Fibrin-based human engineered heart tissue (hEHT) attached to silicon posts (scale bar = 1 mm). **F,G)** Average contraction peaks of hEHTs made from the different HCM isogenic sets, electrically paced at 2 Hz. **H)** hEHTs showed mean contraction forces of 0.070 mN in *MYH7*^WT/WT^, reducing to 0.053 mN in *MYH7*^WT/MUT^, and 0.037 mN in *MYH7*^MUT/MUT^ hiPSC-CMs (*N* = 4–7 independent biological replicates); ACTC1^WT/WT^ hEHTs produced mean forces of 0.050 mN, which increased to 0.080 mN in the ACTC1^WT/MUT^ genotype (N = 3–11 independent biological replicates). **I,J)** Both isogenic sets of mutant EHTs showed significantly prolonged contraction and relaxation times in comparison to their respective isogenic controls. **K,L)** Mitochondrial respiration profiles of both sets of HCM mutants, displaying a higher oxygen consumption rate (OCR) relative to their healthy isogenic counterparts. **M)** Basal respiration rate was higher in *MYH7*^WT/MUT^ (~52% increase) and *MYH7*^MUT/MUT^ (~20%) when compared to WT control (*N* = 12 independent biological replicates), which was also verified in *ACTC1* mutants (~2.6-fold increase vs isogenic WT, *N* = 9 independent biological replicates). **N)** Maximal respiration rate accompanied the increasing trend with the mutation load (~35% higher in heterozygous and ~ 43% higher in homozygous *MYH7*-mutants relative to isogenic WT control; ~93% increase in *ACTC1* mutants vs isogenic WT control). Data: mean ± SD, one-way ANOVA + Newman-Keuls correction for multiple comparisons in *MYH7* isogenic trio and unpaired *t*-test between *ACTC1* lines, *p < .05, **p < .01, ***p < .001, ****p < .0001.

Contractility was assessed in 2D using the CellOPTIQ™ and pixel displacement algorithms [27] to estimate the cell shortening of the different hiPSC-CM lines. This revealed that, while *MYH7*-mutant hiPSC-CMs displayed a hypo-contractile phenotype, *ACTC1*^WT/MUT^ hiPSC-CMs exhibited hyper-contractility relative to their respective isogenic controls (Fig. 3A-D), illustrating a key difference in phenotype between these two HCM-associated mutations.

Analysis of contractile force was further performed using 3D human engineered heart tissues (hEHTs), which enable measurements of force generation by evaluating the movement of silicone posts attached to the tissue [28]. Changes in spontaneous beat rates follow the same trend as in the 2D platform, with only the *MYH7*-mutation increasing the spontaneous beating frequency (**Fig. S4E**). Force generation at 2 Hz electrical pacing was measured in fibrin EHTs made with the different hiPSC-CM lines and corroborated the contrasting hypo- vs hyper-contractile phenotypes seen in *MYH7* and *ACTC1* mutants, respectively (Fig. 3E-H). Interestingly, both mutations led to prolonged contraction and relaxation times in comparison to their isogenic controls (Fig. 3I,J).

### 3.5 Metabolic respiration profiles are similar in both HCM mutations

To further evaluate the impact of the two HCM mutations, energy metabolism of the various lines was investigated using the SeaHorse XF Mito Stress Test assay, to assess mitochondrial respiration. In both isogenic sets of HCM models, presence of a sarcomeric mutation increased the oxygen consumption rate (OCR) during sequential addition of electron transport chain inhibitors (Fig. 3K,L). Indeed, basal and maximal respirations increased significantly in both mutant sets (Fig. 3M,N), supporting the energy depletion model [29] and indicating that inefficient energy metabolism is a phenotype shared by both mutants.

### 3.6 Gene expression analysis uncovers potential molecular determinants underlying contrasting hypertrophic phenotypes

**Fig. 4:**
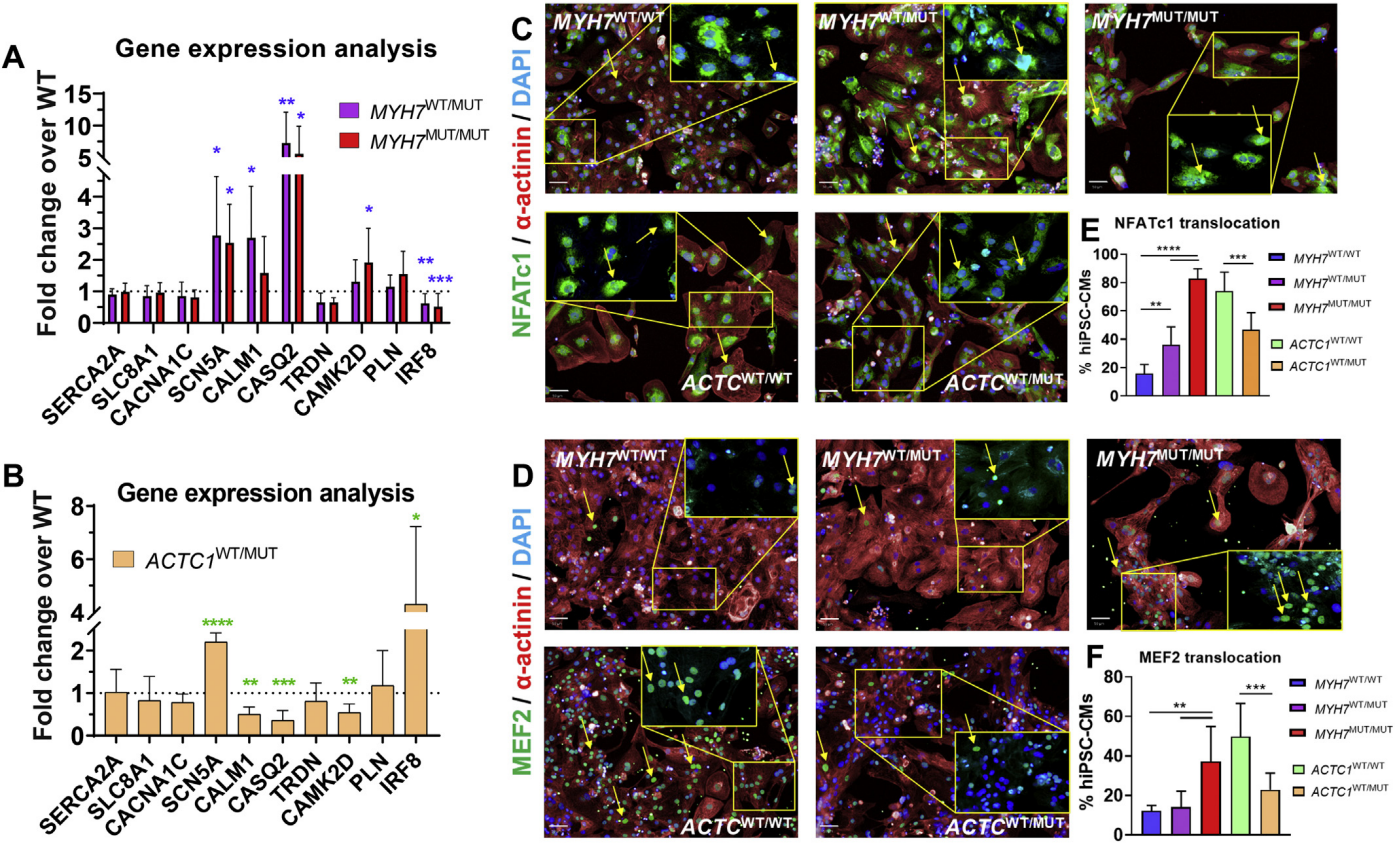
Molecular analysis of calcium handling and hypertrophic pathways activated in HCM hiPSC-CM lines. qRT-PCR analysis of expression of Na^+^ and Ca^2+^ ion channels, Ca^2+^ handling pathways, and their regulators in **A)***MYH7*-mutant lines (*N* = 5–7 biological replicates) and **B)***ACTC1*-mutant hiPSC-CMs (N = 4–6 biological replicates) showed similar upregulation of *SCN5A* in both isogenic collections (~2.6-fold higher in *MYH7*-mutants, ~2.2-fold higher in *ACTC1*-hiPSC-CMs), relative to their respective isogenic WT controls. This also revealed contrasting changes in the expression of Ca^2+^-binding proteins (*CALM1*: ~1.6–2.7-fold higher in *MYH7*-mutants, ~50% lower in *ACTC1*-mutants; *CASQ2*: ~6–7-fold higher in *MYH7*-mutants, ~65% reduced in *ACTC1*-mutants; *CAMK2D*: ~31–91% higher in *MYH7*-mutants, ~46% lower in ACTC1-mutants vs respective isogenic controls), and *IRF8* (~40–50% reduced in *MYH7*-mutants, ~4.3-fold higher in *ACTC1*-mutants vs respective isogenic controls). **C)** Representative fluorescence micrographs highlighting nuclear translocation of NFATc1 and **D)** MEF2 in hiPSC-CMs bearing different genotypes (detailed in respective insets, nuclear-localized transcription factors highlighted by yellow arrows). **E)** Quantification of translocation of NFATc1 and **F)** MEF2 shows increase in the proportion of *MYH7*-mutant hiPSC-CMs displaying nuclear localisation (NFATc1: 2.3-fold higher in WT/MUT and 5.3-fold higher in MUT/MUT; MEF2: 3-fold-higher in homozygous mutant g.MYH7^C9123T^, relative to the isogenic healthy control. *N* = 6 biological replicates). The opposite response was seen in heterozygous *ACTC1* mutants (NFATc1: 37% decrease in nuclear localisation; MEF2: 54% reduction, relative to WT isogenic control, N = 9–12 biological replicates). Data: mean ± SD, one-way ANOVA + Newman-Keuls correction for multiple comparisons in *MYH7* isogenic trio and unpaired *t*-test between *ACTC1* lines, *p < .05,**p < .01, ***p < .001, ****p < .0001. (For interpretation of the references to colour in this figure legend, the reader is referred to the web version of this article.)

In order to uncover disease mechanisms and identify new potential targets for phenotypic rescue of HCM, gene expression analysis was performed using qRT-PCR for selected ion channels, calcium handling pathways and hypertrophy-associated genes (Fig. 4A,B).

These analyses identified an upregulation of the late sodium channel *SCN5A* in both *MYH7-* and *ACTC1-*mutants relative to their respective isogenic controls, suggesting a compensatory response [30]. However, important differences were observed between the two hiPSC-CMs HCM models, with the calcium-binding proteins calmodulin-1 (*CALM1*), calsequestrin-2 (*CASQ2)* and Ca^2+^/calmodulin-dependent kinase II (*CAMK2D*) being upregulated in *MYH7-*mutants and downregulated in *ACTC1-*mutants. Furthermore, *IRF8*, a negative regulator of NFATc1 translocation to the nucleus that is associated to a pathological response [31], was downregulated in *MYH7*-mutants and upregulated in *ACTC1*-mutants.

In order to ascertain whether these gene expression changes were impacting on known hypertrophic signaling pathways, the nuclear translocation of NFATc1 [32] and MEF2C [33] were investigated by high-content imaging **(**Fig. 4C-F**)**. Remarkably, the two mutant lines showed a contrasting pattern: *MYH7*-mutant hiPSC-CMs exhibited an increase in the nuclear translocation of both transcription factors relative to the isogenic control, with the *ACTC1* mutants displaying the opposite response.

### 3.7 Calcium sensitivity of *ACTC1-* or *MYH7*-mutant hiPSC-CM lines identifies potential therapeutic targets for HCM

Despite showing similar arrhythmic responses to the sarcomeric mutations, the two HCM models differed considerably in expression levels of Ca^2+^-binding proteins. Therefore, and given that increased myofilament calcium sensitivity is often detected in HCM models [11,13,34], we aimed to characterize the influence of extracellular Ca^2+^ in the arrhythmogenic phenotypes observed in isogenic hiPSC-CMs, via confocal line scanning.

**Fig. 5:**
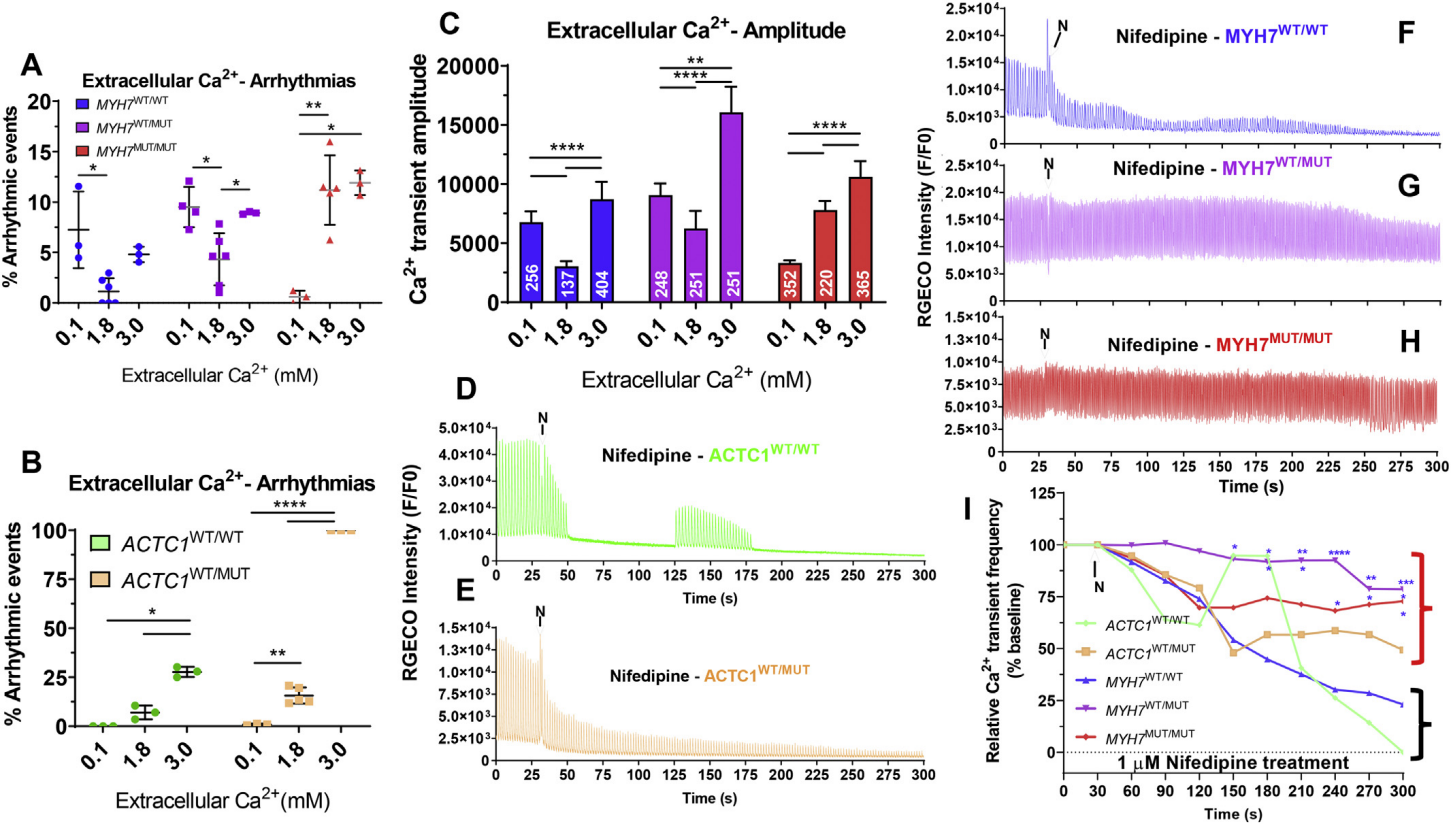
Calcium sensitivity responses of HCM hiPSC-CM lines. Confocal line scanning of **A)***g.MYH7*^*C9123T*^ isogenic trio and **B*)****c.ACTC1*^*G301A*^ mutant vs isogenic healthy hiPSC-CMs exposed to varying concentrations of extracellular Ca^2+^ (*N* = 3–5 biological replicates) showed that the arrhythmogenic phenotype is exacerbated by higher concentration of Ca^2+^ in the medium (3 mM) in all genotypes (~2–4-fold higher frequency of abnormal events in *MYH7-*mutant lines; ~3–6-fold increase in *ACTC1-*mutant hiPSC-CMs). A reduction in the extracellular Ca^2+^ to 0.1 mM reversed this effect in *ACTC1*-mutant lines and in the homozygous g.MYH7^C9123T^ variant. **C)** Changes in the frequency of arrhythmogenic events were in line with variations of amplitude in *MYH7*-mutant hiPSC-CMs. **D-I)** Treatment with 1 μM Nifedipine caused fast cessation of beating in healthy hiPSC-CM lines (black bracket) whereas HCM cells (red bracket) maintained calcium transients for longer (% calcium transient peaks detected after 300 s treatment relative to baseline of 26.1% in MYH7^WT/WT^ vs 78.6% in MYH7^MUT/WT^ and 72.8% in MYH7^MUT/MUT^, N = 5 biological replicates; 0% in ACTC^WT/WT^ vs 49.3% in ACTC^WT/MUT^, N = 3 biological replicates. Data: mean ± SD, number of technical replicates indicated in respective bars in C, one-way ANOVA + Newman-Keuls correction for multiple comparisons in *MYH7* isogenic trio and unpaired t-test between *ACTC1* lines, *p < .05,**p < .01, ***p < .001, ****p < .0001. (For interpretation of the references to colour in this figure legend, the reader is referred to the web version of this article.)

By increasing the extracellular Ca^2+^ from 1.8 to 3.0 mM, the frequency of arrhythmic events rose in all the cellular lines (Fig. 5A,B). However, the decrease of extracellular Ca^2+^ to 0.1 mM triggered different responses: arrhythmias were nearly abolished in both *ACTC1* sets but this was only true in the homozygous variant of the *MYH7-*mutant hiPSC-CMs. The changes in arrhythmogenicity of the *MYH7* lines were in line with the variation of calcium transient amplitude (Fig. 5C). Altogether, these results indicated that, by limiting the amount of Ca^2+^ available for contraction, the arrhythmogenic phenotype could potentially be reversed in some sarcomeric mutant hiPSC-CMs.

To further demonstrate that HCM hiPSC-CMs displayed perturbed calcium homeostasis underlying arrhythmogenic phenotypes, pharmacological antagonism of the L-type calcium channel by the addition of 1 μM nifedipine was performed during confocal laser line scan experiments. Both *MYH7-* and *ACTC1*-mutant hiPSC-CMs were capable of maintaining contraction upon 1 μM nifedipine addition, whereas the respective wild-type isogenic controls slowed or stopped beating altogether after L-type calcium channel blockade (Fig. 5D-I), as expected [35].

### 3.8 Rescue of arrhythmogenic phenotypes with pharmacological intervention

High intracellular calcium, particularly during diastole, is known to induce arrhythmias and abnormal calcium transient events [36]. It can also drive the electrogenic Na^+^/Ca^2+^ exchanger (NCX), resulting in further inward flow of Na^+^ [37]. Given that cytosolic Ca^2+^ levels interfered with the magnitude of the arrhythmic phenotype, coupled with the transcriptomic changes in genes encoding sodium channel (*SCN5A*) and calcium-binding proteins, calcium handling pathways were targeted by pharmacological approaches.

Dantrolene is a ryanodine receptor antagonist that inhibits sarcoplasmic Ca^2+^ release into the cytosol [38], and thus attenuates cytosolic Ca^2+^ build-up in hiPSC-CMs upon depolarization. Treatment of HCM lines with 10 μM dantrolene resulted in a modest reduction in the arrhythmogenic events (**Fig. S5A,B**), evaluated by confocal line scanning.

**Fig. 6:**
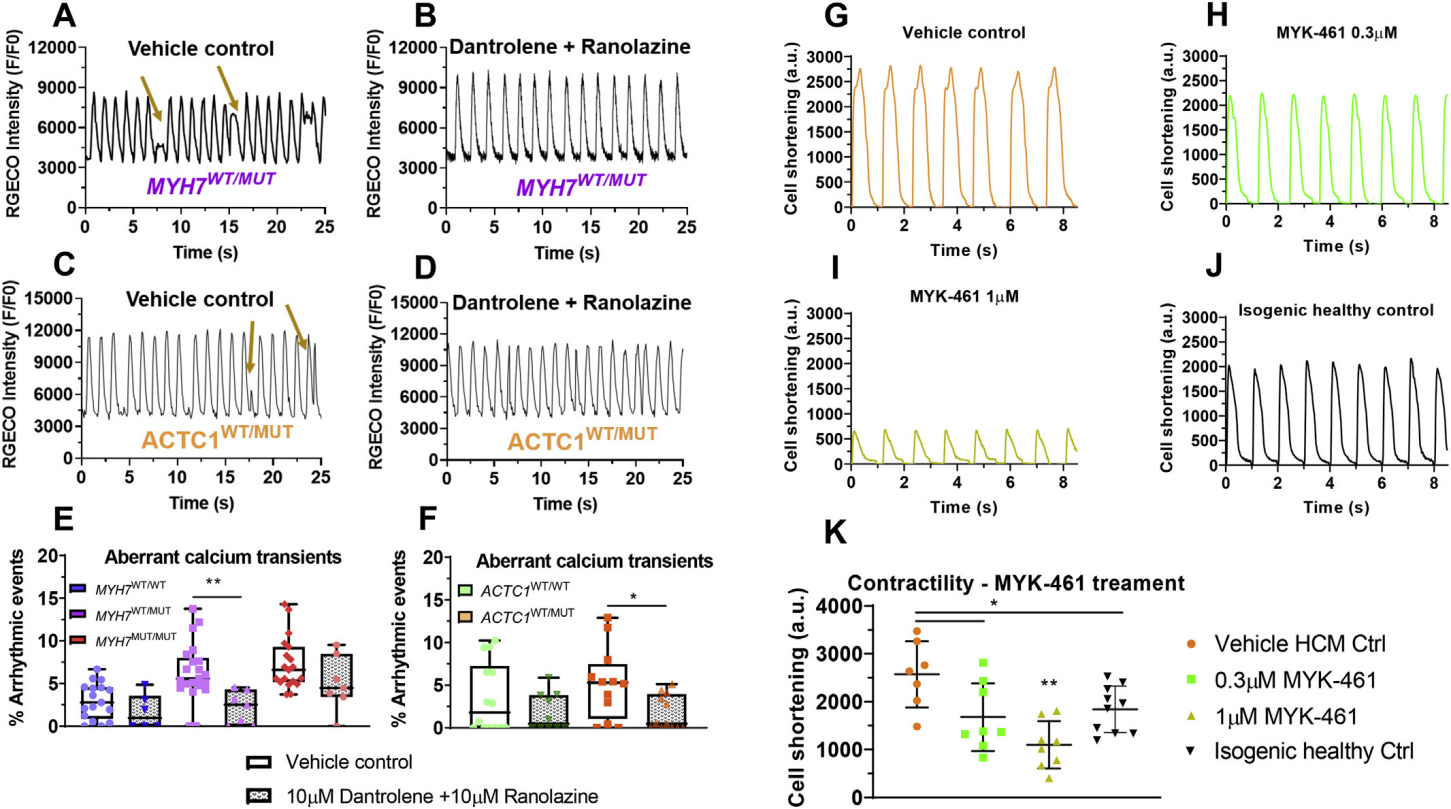
Pharmacological intervention strategies to rescue HCM phenotypes. Representative confocal line scans of heterozygote g.*MYH7*^C9123T^ hiPSC-CMs treated with **A)** 0.1% (*v*/v) DMSO (vehicle control) or **B)** 10 μM dantrolene +10 μM ranolazine, with **C,D)** referring to c.*ACTC1*^G301A^ hiPSC-CMs in the same respective order. **E)** Quantification of aberrant calcium transients (golden arrows) in *MYH7* isogenic trio showed reduction of frequency of arrhythmogenic events in heterozygote mutants back to healthy control upon treatment with dantrolene + ranolazine (2.9-fold decrease relative to vehicle control, *N* = 7–20 biological replicates). **F)** The combination treatment also reduced the frequency of abnormal calcium transients in *ACTC1*^WT/MUT^ to levels similar to healthy isogenic control (3-fold reduction vs vehicle control, *N* = 10–12 biological replicates). Representative contractility traces of optically-paced (1 Hz) ChR2^+^*c.ACTC1*^G301A^ hiPSC-CMs treated with **G)** 0.1% (v/v) DMSO (vehicle control), **H)** 0.3 μM MYK-461 and **I)** 1 μM MYK-461, relative to **J)** WT isogenic control. **K)** Quantification of contractility in 2D CellOPTIQ™ shows that treatment with MYK-461 rescues hyper-contractile phenotype of *ACTC1*-mutant hiPSC-CMs back to isogenic healthy levels (35–57% decrease in contractility relative to vehicle control, *N* = 8 biological replicates). Data: box and whisker plots or mean ± SD, drug treatments were compared to vehicle controls using unpaired Student's t-test, *p < .05, **p < .01, ***p < .001, ****p < .0001.

Ranolazine is an enhancer of the outward mode of NCX, by blocking late sodium current and subsequently promoting intracellular Ca^2+^ efflux [37]. While this drug showed partial efficiency in isolated mouse cardiomyocytes [39]*,* clinical trials have not demonstrated functional efficacy in HCM patients [37,40]. Thus, a combination treatment of 10 μM dantrolene and 10 μM ranolazine was performed on the isogenic HCM hiPSC-CM lines. This strategy proved effective in reducing the frequency of arrhythmic events in heterozygous mutants (representative of HCM patient genotypes) to levels comparable to their respective isogenic healthy controls (Fig. 6A-F).

### 3.9 Targeting opposing HCM phenotypes with contractility modulators

While both isogenic sets displayed similar arrhythmogenic phenotypes, changes in contractility were diametrically opposed, with *MYH7*-mutants displaying hypo-contractility and *ACTC1*-mutants exhibiting a hyper-contractile phenotype.

Omecamtiv mecarbil (OM) prolongs the binding of myosin heads to actin by increasing the speed of ATP hydrolysis, thereby enhancing cardiac contractility [41], and is showing promising results in ongoing phase II clinical trials [42]. However, treatment of *MYH7*-mutant hiPSC-CM lines has not resulted in any functional reversal of the hypo-contractile phenotype shown (**Fig. S5C-J**), as assessed by 2D pixel-displacement contractility measurements under 1 Hz pacing.

In contrast, mavacamten (also known as MYK-461) is a cardiac myosin ATPase inhibitor that counteracts hyper-contractility, as demonstrated in a mouse model of HCM [43], which translated in a reduction of LVOT obstruction and improved exercise capacity in patients [44]. Treatment of p.ACTC1-E99K hiPSC-CMs with 0.3 μM MYK-461 led to reversal of the hyper-contractile phenotype towards the levels seen in the wild-type isogenic counterparts (Fig. 6G-K).

Taken together, these approaches illustrate the complexity and phenotypic variability between the two HCM-associated mutations. Nonetheless, bespoke pharmacological intervention was able to ameliorate key phenotypes, with combination treatment successfully rescuing arrhythmogenicity in both models.

## 4 Discussion

HCM is a prevalent and complex genetic disease, with highly variable clinical presentation and outcomes [3]. Due to the numerous mutations associated with this condition, phenotype severity, expressivity and penetrance differs greatly both within and between different genetic variants. Here, we described the phenotypic and mechanistic similarities and differences between two HCM-associated mutations - g.*MYH7*^C9123T^ and c.*ACTC1*^G301A^, by comparing isogenic sets of hiPSC-CMs to provide physiologically-relevant in vitro models. The use of the isogenic approach minimizes the baseline variability of hiPSC-CMs features due to unrelated genetic backgrounds (as detected by changes in baseline contraction force in 3D hEHTs [15], or differences in the basal and maximal respiration levels between healthy lines). This approach allows focus to be centred on the influence of each mutation on disease progression [21]. While patients typically show heterozygous mutations in sarcomeric genes [45] (recaptured in the hiPSC-CM lines), the use of *MYH7* homozygous-mutant lines for in vitro disease modeling provides extra readout sensitivity for the phenotypic assays developed. Moreover, by applying the same experimental conditions to all the lines in parallel, such as defined serum-free medium and pacing, the impact of underlying biological variability between isogenic lines (e.g., different beat rate) has been minimized [46].

We utilized the GECI R-GECO1.0 to image calcium transients in hiPSC-CMs and identified an association between aberrant events and mutation load in both isogenic sets. Commercially available calcium dyes typically exhibit gradual dye extrusion, which limits the time-frame of imaging experiments and may lead to experimental artefacts due to undesired compartmentalization in the endoplasmic reticulum [47]. In contrast, the use of a genetic calcium sensor provided experimental flexibility, and alleviated the problems of dye leakage and increased toxicity experienced when using traditional calcium indicator chemical dyes [48,49]. This GECI was inserted into the extensively studied safe locus *AAVS1*, whereby intronic disruption of the *PPP1R12C* gene has not reported to cause deleterious cellular effects [24]. Importantly, while an increase in diastolic calcium and overall intracellular calcium overload (as evidenced by response to nifedipine) was seen in both models, a higher systolic calcium peak amplitude was only observed in *MYH7-*mutant hiPSC-CMs. Interestingly, recent reports using cytoplasmic R-GECO1.0 to study the p.R92Q-cTnT and p.R145G-cTnI HCM-associated mutations found systolic peak amplitude changes in the latter model but not the in the former [50]. Thus, genetically-encoded calcium reporter cell lines are increasingly useful tools to investigate discrepancies of calcium transient abnormalities in HCM patients bearing different mutations [51]. Finally, both HCM models did not reveal differences in SR Ca^2+^storage as sarcomeric mutant hiPSC-CMs showed similar responses to caffeine as healthy lines. These data are further supported by the lack of changes in expression of genes involved in SR Ca^2+^release (TRDN, PLN) or re-uptake (SERCA2A). Remarkably, this response contrasts with the p.R442-βMHC hiPSC-CMs that showed depressed SR Ca^2+^storage which is accompanied by downregulation of SERCA2A and RYR2 [13], further strengthening the notion that HCM effects are mutation-specific.

One of the most striking differences between the p.β-MHC-R453C and p.ACTC1-E99K hiPSC-CM models was altered contractility. g.*MYH7*^C9123T^ mutants exhibited hypo-contractility, which contrasts to the hyper-contractility phenotype reported in C_2_C_12_ murine myoblasts forced to overexpress recombinant β–MHC with the same mutation [18]. This highlights potential species-differences, particularly in terms of isoform expression, as rodent hearts exhibit different ventricular predominance of α vs β-MHC expression relative to humans [52,53]. Nevertheless, even in other hiPSC-CM models, the p.β–MHC-E848G mutation was found to be hypo-contractile [17], whilst the p.β–MHC-R403Q mutation was found to be hyper-contractile [20]. Altogether, the assertion that all thick filament mutations behave similarly must be challenged. Importantly, our data is in line with direct myofilament evaluation studies performed in human cardiomyocytes bearing several *MYH7* mutations, whereby a loss in maximal contraction force is not accompanied by changes in Ca^2+^sensitivity [16]. In contrast, p.ACTC1-E99K mutant hiPSC-CMs revealed hyper-contractility. This tallies with existing mouse models of this mutation, which show a 3–4-fold greater force generation compared to a non-transgenic control [54]. Notably, both mutations caused a similar increase in both contraction and relaxation times, detected in 3D hEHTs.

The energy depletion model, which states that sarcomere mutations cause inefficient ATP usage, thereby increasing the energetic demand on cardiomyocytes, is regarded as the main source of myocardial energy deficiency [29]. This often results in sudden cardiac death particularly in athletes, where a fast-beating heart necessitates even more energy. We have observed a greater mitochondrial respiration rate in diseased lines relative to their isogenic healthy controls, which is characteristic of the compensatory stage of HCM that precedes energy depletion. Thus, we postulate that this inefficient ATP usage impinges on the ability of SERCA2A (whose expression levels are unaltered relative to healthy isogenic controls, indicating a lack of compensation) to pump Ca^2+^ back into the sarcoplasmic reticulum during diastole. This in turn leads to high intracellular calcium and triggered aftercontractions, detected as calcium transient abnormalities [13,36].

**Fig. 7:**
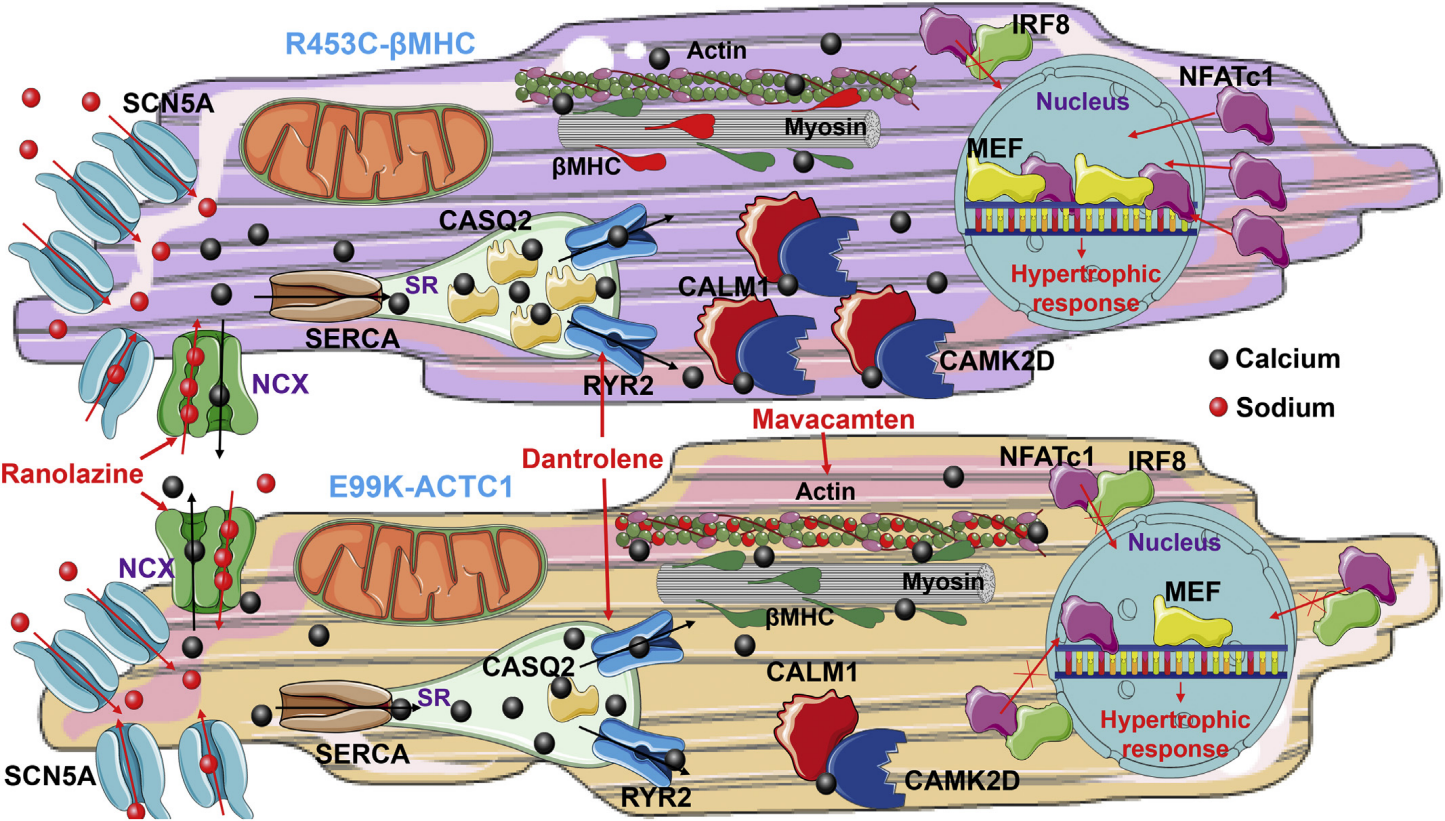
Mutation-specific disease mechanisms and pharmacological rescue of HCM. Both hiPSC-CM models shared upregulation of SCN5A and energy depletion as HCM mechanisms, which may impair Ca^2+^ re-uptake into the sarcoplasmic reticulum (SR) and precipitate arrhythmias. The R453C-βMHC model showed up-regulation of CASQ2, CALM1 and CAMK2D and increased cytosolic Ca^2+^ levels, which further contribute to the arrhythmogenic phenotype. IRF8 was downregulated, promoting MEF and NFATc1 nuclear translocation to trigger hypertrophic response. Contrastingly, the E99K-ACTC1 model showed the opposite gene expression profile. The arrhythmogenic phenotype was rescued in both models by combination treatment with ranolazine and dantrolene. The hypercontractility displayed in E99K-ACTC1 cardiomyocytes was rescued by mavacamten. SR, Sarcoplamic reticulum; RYR2 – Ryanodine receptor; NCX, sodium‑calcium exchange pump; SCN5A – sodium channel; CASQ2 – calsequestrin; CALM1 – calmodulin; CAMK2D - Ca^2+^/calmodulin-dependent kinase II.

Calcium is known to play a key role in hypertrophic signaling, via calcineurin-mediated activation of NFATc1 [32] and MEF2C [33], resulting in their translocation to the nucleus where hypertrophic gene pathways (e.g., fetal gene program [55]) are initiated. Despite converging on calcium transient disruption as a key hallmark of HCM progression (leading to the observed arrhythmias and prolonged beating upon nifedipine treatment), the two models differ greatly in the expression of Ca^2+^-binding proteins and subsequent hypertrophic transcription factor activation. Remarkably, *MYH7*-mutant hiPSC-CMs displayed up-regulation of *CALM1*, *CASQ2* and *CAMK2D* relative to their healthy isogenic controls, and a reduction in expression of *IRF8* (a negative regulator of NFATc1 nuclear translocation [31]), with the opposite being shown in *ACTC1*-mutants. This led to contrasting profiles of nuclear translocation of NFATc1 and MEF2 between the two isogenic sets. We reason that the transcriptomic discrepancies between the two HCM models underlie this difference, as by controlling the levels of calsequestrin [56], calmodulin [57] and consequently intracellular Ca^2+^ available, the activation of transcription factors like IRF8, NFATc1 and MEF2 is affected, affecting downstream hypertrophic pathways (Fig. 7). Moreover, elevated Ca^2+^/calmodulin-dependent kinase II (*CAMK2D*) activity has shown to trigger arrhythmogenesis by phosphorylating ryanodine receptor, which leads to increased SR Ca^2+^ release [58,59]. CAMK2D also promotes MEF2 translocation to the nucleus by phosphorylating the MEF repressor HDAC4, promoting its nuclear export [60]. Importantly, the up-regulation of *CAMK2D* in *MYH7*-mutant hiPSC-CMs is in line with the higher levels of cytosolic Ca^2+^ detected, and increased nuclear translocation of MEF2 in these cells, with the *ACTC1*-mutant cardiomyocytes showing a consistently opposite profile.

We speculate that these transcriptomic changes are akin to gene modifiers that contribute to the magnitude of the HCM phenotypes elicited by the primary sarcomeric mutation [61]. The c.*ACTC1*^G301A^ variant is particularly interesting in that individuals with the same mutation often display a wide variety of phenotypic severity [62,63]. Indeed, our own studies on a family harbouring this mutation showed differences in clinical and phenotypic features, depending on the individual under study [19]. These differences could be the result of a myriad of factors including age, expression of disease-modifying genes, epigenetics modifiers, miRNAs and lncRNAs [61,64]. The use of large-scale transcriptomic analyses from isogenic sets of hiPSC-CMs [65] may uncover new drug targets, including gene modifiers who remain unexplored.

Nevertheless, we harnessed the mechanistic data generated to guide pharmacological intervention strategies. In order to counter the elevated concentration of intracellular Ca^2+^, dantrolene was the drug of choice due to its success in rescuing aberrant Ca^2+^ leakage in a mouse model of troponin-T HCM, by stabilizing Ca^2+^ release from hyper-phosphorylated ryanodine receptors [66]. However, in both HCM hiPSC-CM models, the anti-arrhythmogenic effect of dantrolene was mild. Importantly, *SCN5A* was upregulated in both sarcomeric mutants, in comparison to their respective isogenic controls. This may be a compensatory mechanism for irregular NCX activity, or as a result of phosphorylation by CaMKIIδc [67]. Nonetheless, this increased *SCN5A* expression has also been observed in a hiPSC-CM model of c.*MYH7*^C1324G^ mutation [13]. We therefore chose ranolazine as a late sodium channel antagonist, a drug which has been shown to reverse NCX dysfunction and reduce the occurrence of delayed afterdepolarisations that occurred in a range of human ex vivo HCM heart samples [37]. As ranolazine failed to show full clinical efficacy [40], we combined it with dantrolene, resulting in the abrogation of the arrhythmogenic phenotype of heterozygous mutant HCM hiPSC-CMs, in both models.

Contractility measurements based on pixel displacement of hiPSC-CM monolayers corroborated the phenotypes shown in more physiologically-relevant 3D hEHTs. Thus, we used the 2D format to test the effect of several compounds, due to its higher throughput and lower cell requirement [68]. These modulators of contractility displayed a mixed success, with mavacamten restoring the hyper-contractile phenotype of *ACTC1*-mutant hiPSC-CMs, but OM lacking functional recovery of hypo-contractility shown by *MYH7*-mutant cardiomyocytes. It is worth noting that the effects of OM are dependent on cytosolic Ca^2+^ levels, enhancing contractile force at lower concentrations, with the opposite effect at higher ones, as reported in skinned cardiomyocytes [69]. Given the observed increase in intracellular Ca^2+^ in *MYH7*-mutant lines, it is thus likely that OM is favouring a reduction in contractility, thus failing to rescue the phenotype. Alternatively, the high pacing applied (required due to intrinsically high beat rates of hiPSC-CMs) may mask the positive inotropic effects of OM. Translating the success demonstrated by mavacamten to hEHT format is the goal of future focused studies, incorporating several hypercontractile-causing HCM mutations. Moreover, while further insight may be gained by inferring causality from the pharmacological treatment across phenotypes, these experiments are technically very challenging, e.g., due to differences in time scale between direct drug effects and indirect molecular signaling pathways or cellular responses. This will require further optimization across several orders of magnitude of drug concentrations and/or adaptation of existing phenotypic platforms [27].

In conclusion, we show that sarcomeric mutations leading to HCM can vary greatly in phenotype and mechanism. Each genetic variant must therefore be evaluated individually in order to identify more effective treatments. Our results indicate that future in vitro, mechanistic-driven drug screens may benefit from combinatorial drug treatments to relieve mutation-specific phenotypes.

## 5 Limitations

HiPSC-CMs are known to be immature in terms of ion expression, sarcomere alignment and other ultrastructural differences, relative to adult cardiomyocytes [68,70]. These factors may impact the phenotypes of the disease models studied. Several maturation strategies under development are aimed at improving maturation by several approaches including chronic pacing [71] and biochemical cues [72]. Expectedly, this will generate more robust disease models to study human cardiac disease in vitro. Nevertheless, it is reassuring that the outcomes in our data were consistent between 2D and 3D (despite the relatively low number of biological replicates performed in the latter format), with hiPSC-CMs fabricated into hEHTs known to be of a higher maturity status [73]. Overall, while hiPSC-CMs do not fully represent the complex architecture, multicellularity and neuro-hormonal control of the adult heart, they can be used to model genetically-complex diseases such as HCM in order to uncover new mechanistic information that drives the further development of efficient therapies, as shown herein.

The following are the supplementary data related to this article

Supplementary material 1 Image 1

Supplementary material 2 Supplementary material

Supplementary Table 1 Primers used for PCR genotyping and Sanger sequencing genome-edited hiPSCs. Supplementary Table 1

Supplementary Table 2 Taqman® (Applied Biosystems) probes used for qRT-PCR. Supplementary Table 2

Supplementary Video 1 Confocal line scanning showing spontaneously beating hiPSC-CMs expressing RGECO1.0. Supplementary Video 1

## Funding

This research was funded by the British Heart Foundation (BHF) [grant numbers: SP/15/9/31605, PG/14/59/31000, RG/14/1/30588, RM/13/30157, P47352/CRM], Britain Israel Research and Academic Exchange Partnership (BIRAX) [04BX14CDLG], Medical Research Council (MRC) [MR/M017354/1], Engineering and Physical Sciences Research Council (EPSRC) [DM's Doctoral Prize Research Fellowship], National Centre for the Replacement, Refinement, and Reduction of Animals in Research (NC3Rs) [CRACK-IT:35911–259146, NC/K000225/1, NC/S001808/1], Heart Research UK, German Research Foundation [DFG-Es-88/12–1, HA3423/5–1], European Research Council [ERC-AG-IndivuHeart], European Commission [FP7- Biodesign], German Centre for Cardiovascular Research (DZHK), German Ministry of Education and Research, and the Freie und Hansestadt Hamburg.
