# Supplementary material for: Isogenic models of hypertrophic cardiomyopathy unveil differential phenotypes and mechanism-driven therapeutics
Source: J Mol Cell Cardiol. 2020 Aug;145:43–53. doi: 10.1016/j.yjmcc.2020.06.003 (PMC7487780; doi:10.1016/j.yjmcc.2020.06.003)
Supplement: Supplementary file 1 — Supplementary material 1 [file mmc1.pptx]

## Slide 1
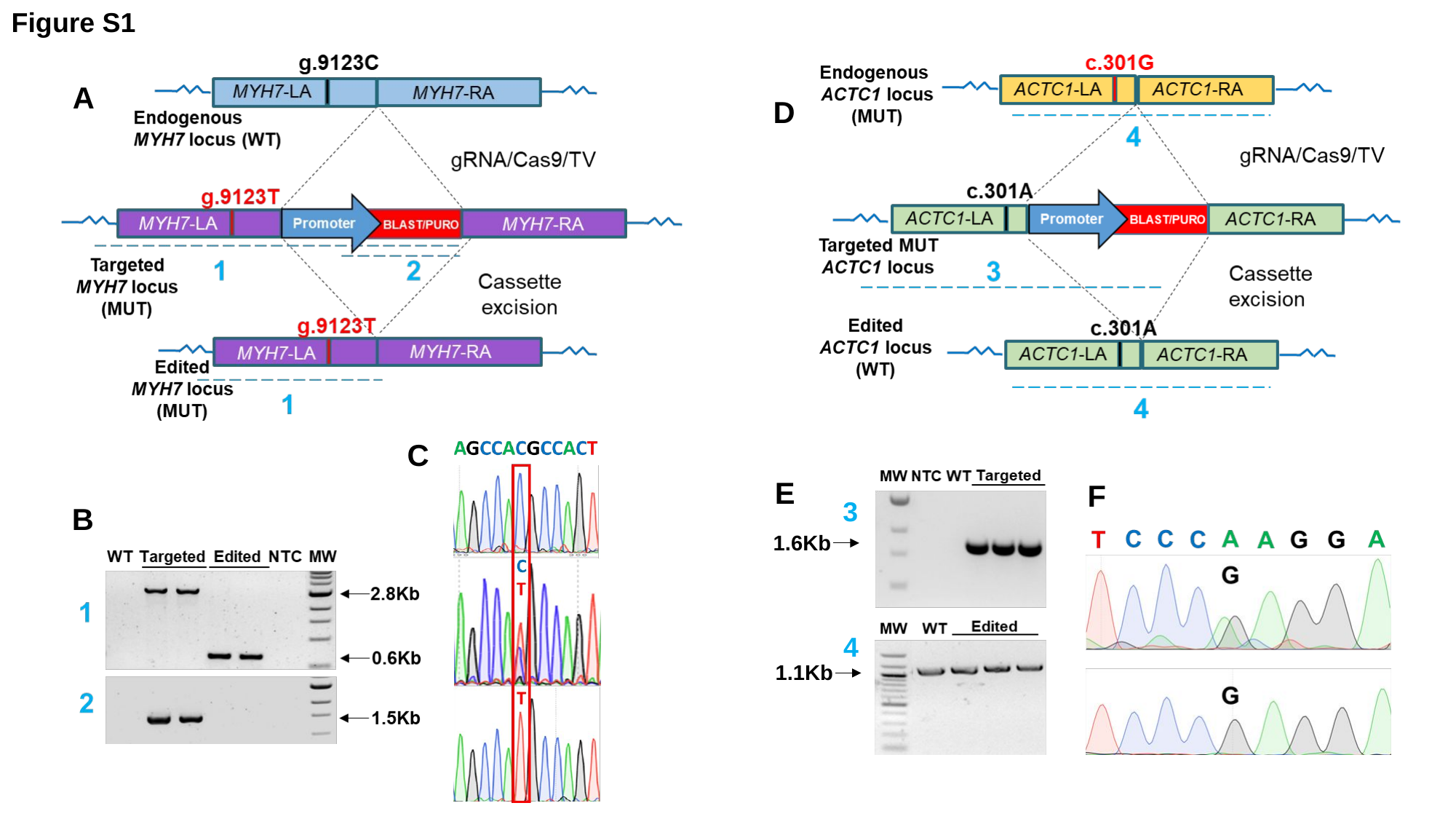

Figure S1
A
D
C
E
F
3
B
1.6Kb
4
1.1Kb

## Slide 2
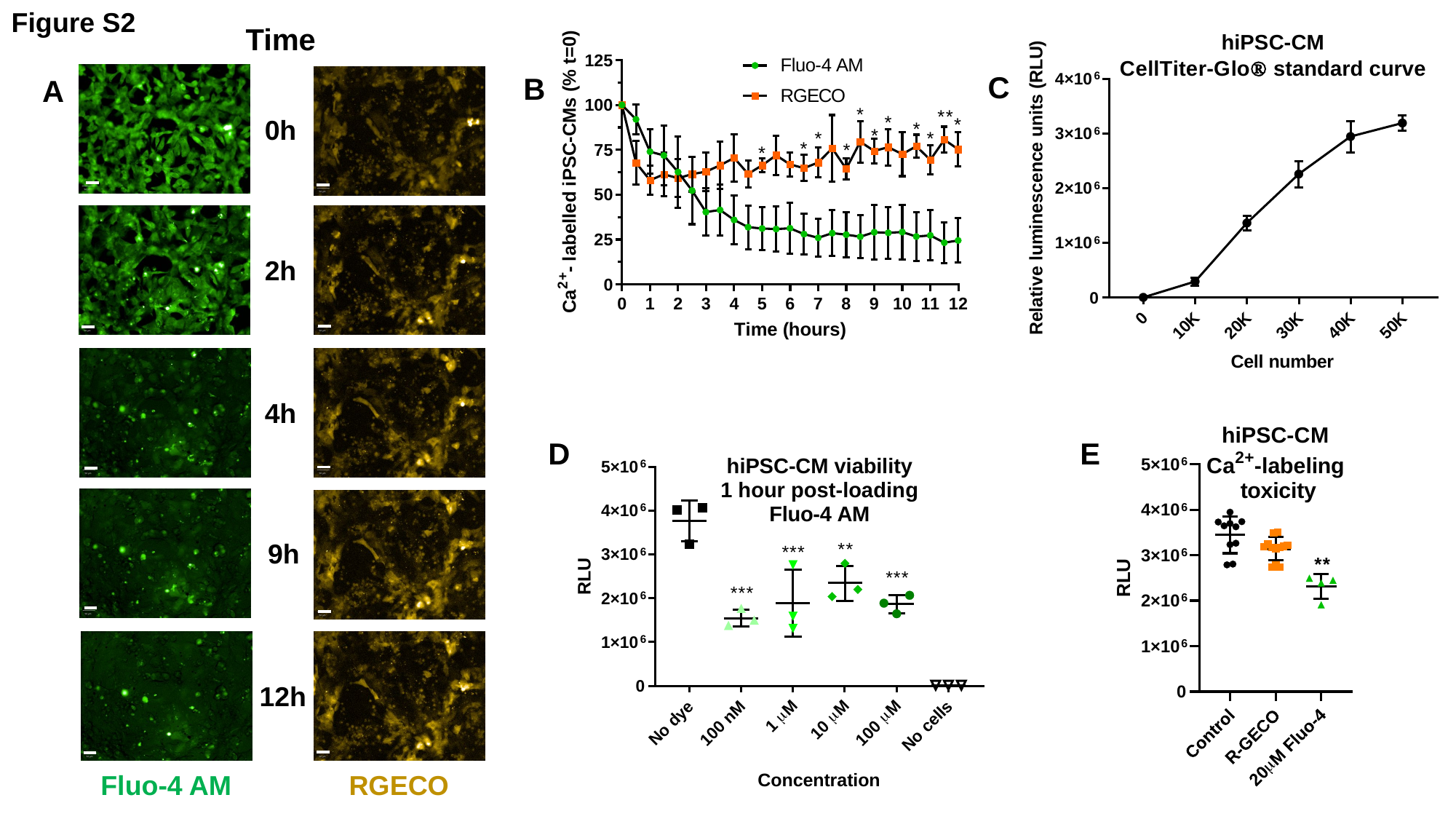

Figure S2
Time
0h
2h
4h
9h
12h
C
B
A
D
E
Fluo-4 AM
RGECO

## Slide 3
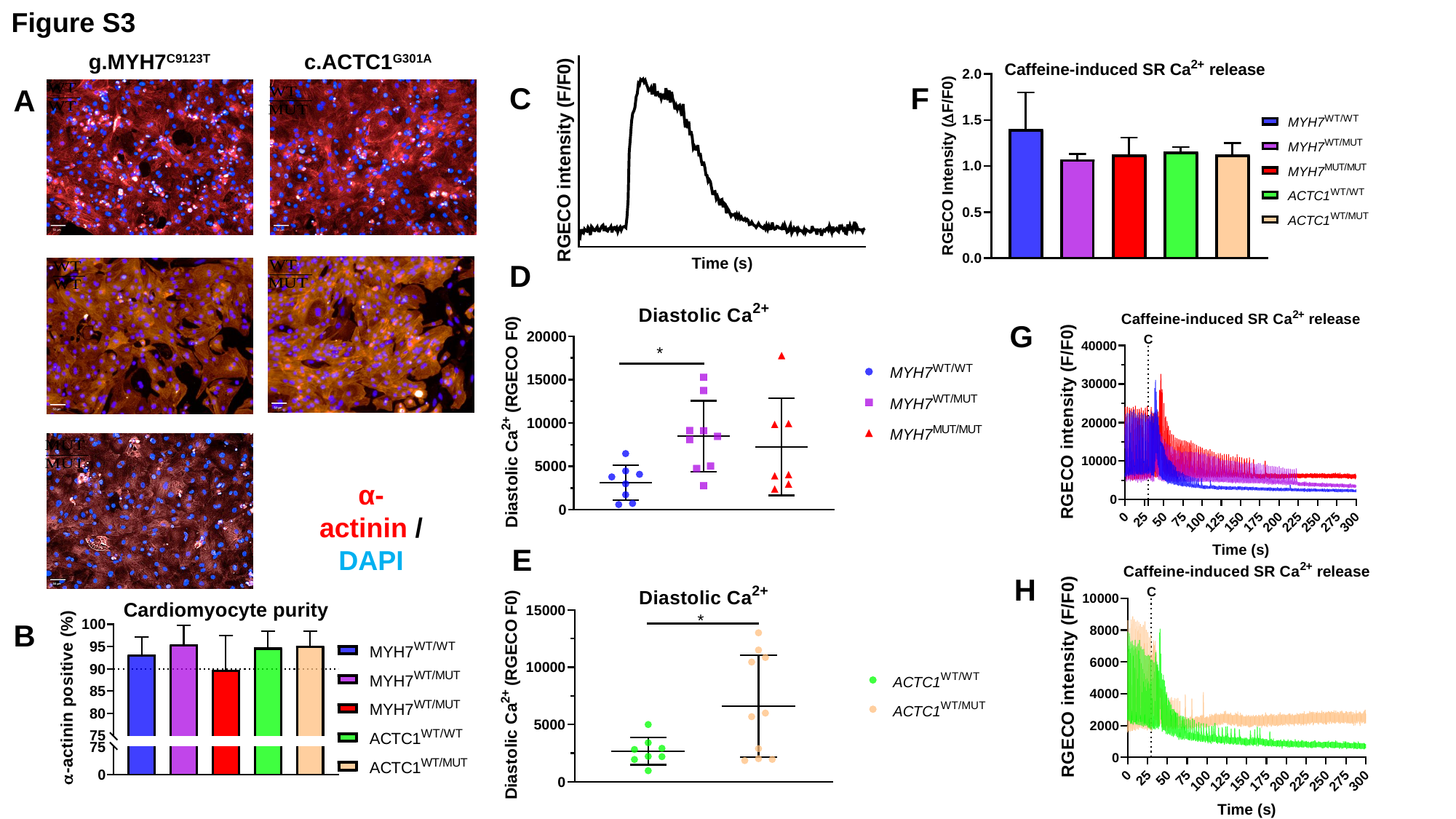

Figure S3
g.MYH7C9123T
c.ACTC1G301A
α-actinin / DAPI
C
F
A
D
G
E
H
B

## Slide 4
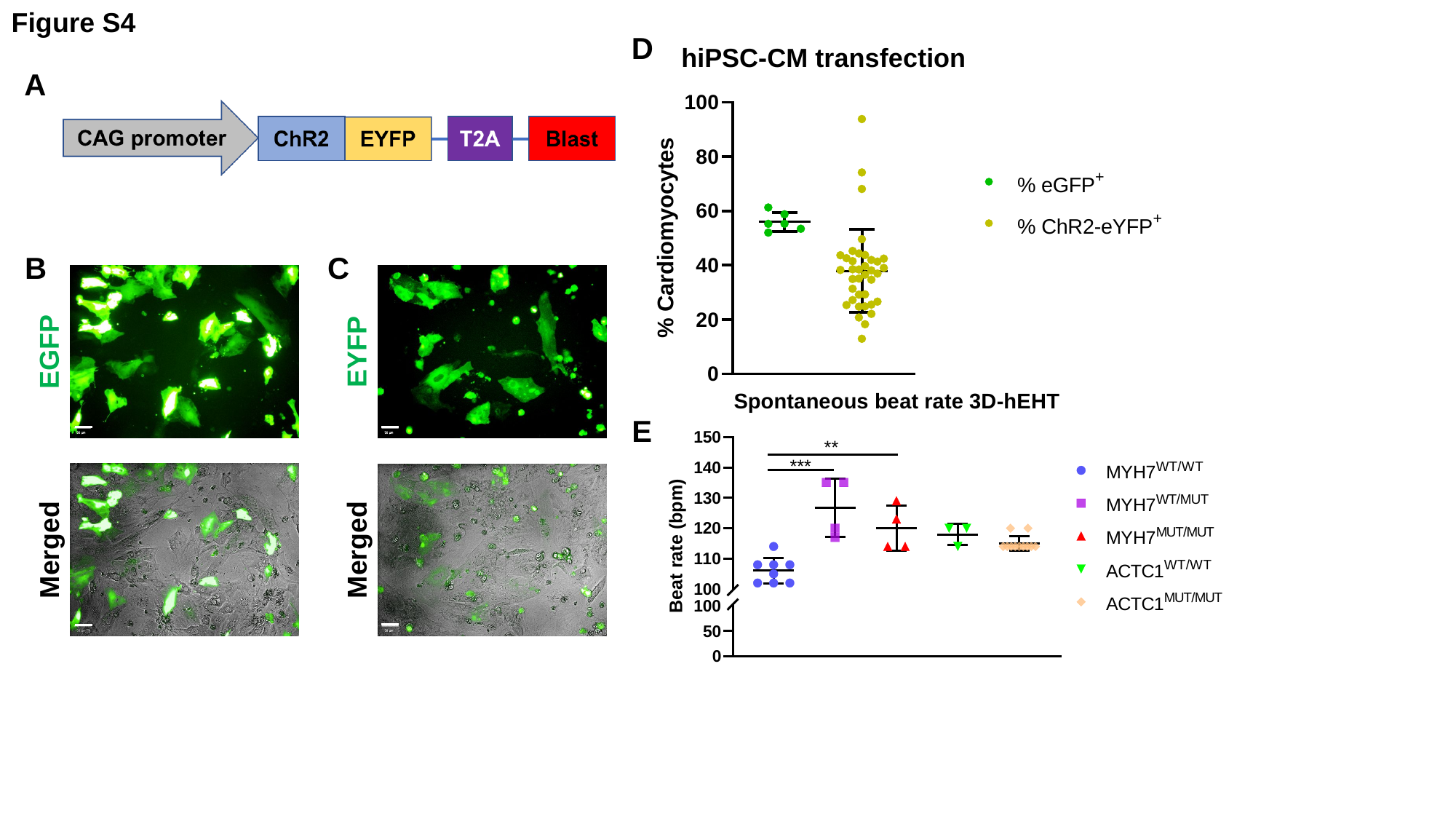

Figure S4
D
A
B
C
EGFP
EYFP
Merged
Merged
E

## Slide 5
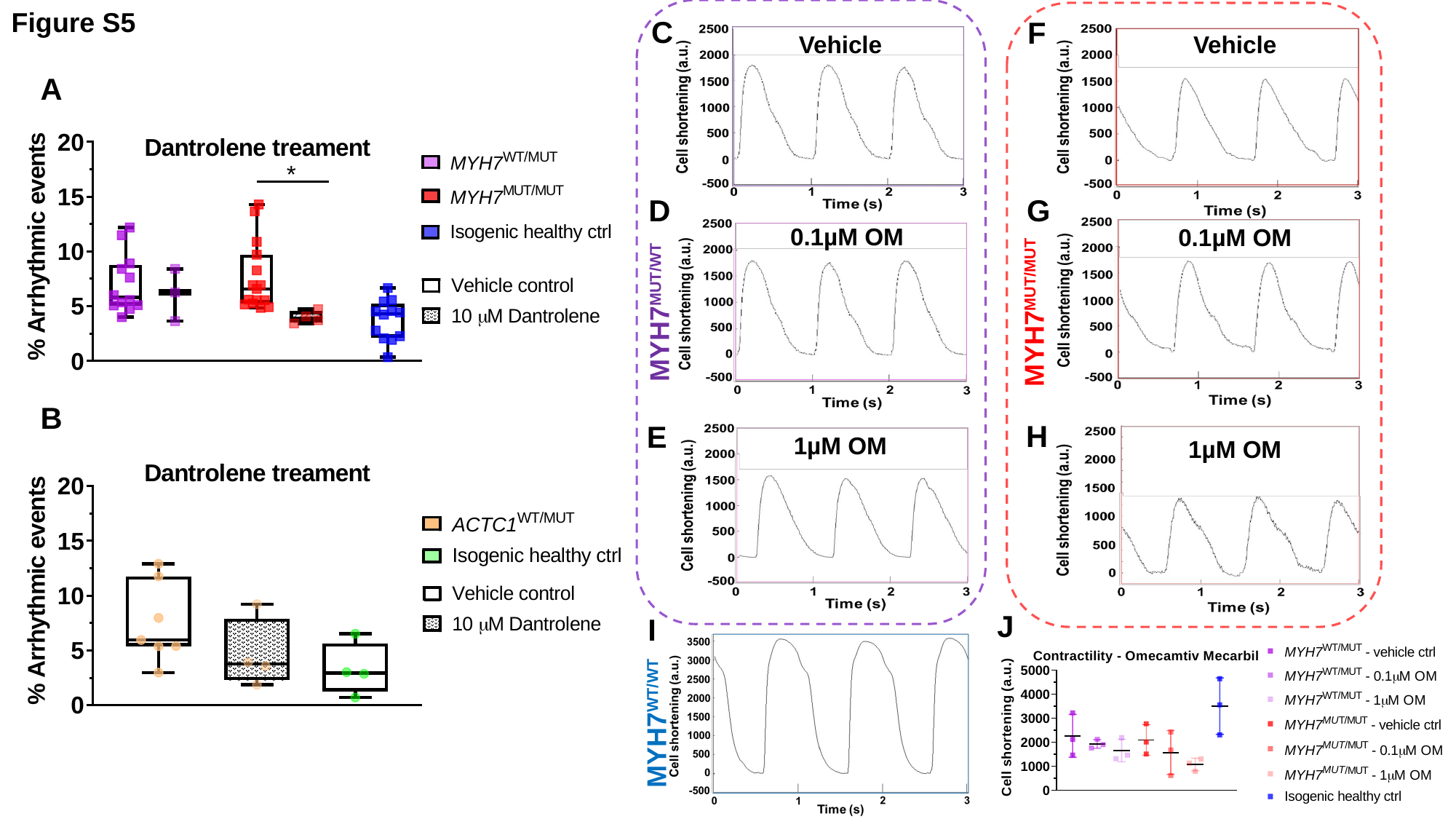

Figure S5
C
F
Vehicle
Vehicle
A
D
G
0.1µM OM
0.1µM OM
MYH7MUT/MUT
MYH7MUT/WT
B
H
E
1µM OM
1µM OM
J
I
MYH7WT/WT
