# Supplementary material for: Isogenic models of hypertrophic cardiomyopathy unveil differential phenotypes and mechanism-driven therapeutics
Source: J Mol Cell Cardiol. 2020 Aug;145:43–53. doi: 10.1016/j.yjmcc.2020.06.003 (PMC7487780; doi:10.1016/j.yjmcc.2020.06.003)
Supplement: Supplementary file 2 — Supplementary material 2 [file mmc2.docx]

**Supplementary Material**

**Isogenic models of hypertrophic cardiomyopathy unveil differential phenotypes and mechanism-driven therapeutics**

Jamie R. Bhagwan^a,1,^*, Diogo Mosqueira^a,1,^*, Karolina Chairez-Cantu^a^, Ingra Mannhardt^b^, Sara E. Bodbin^a^, Mine Bakar^a^, James G.W. Smith^a,c^, and Chris Denning^a,^*

^a^Division of Cancer & Stem Cells, Biodiscovery Institute, University of Nottingham, NG7 2RD, UK. ^b^Department of Experimental Pharmacology and Toxicology, Cardiovascular Research Center, University Medical Center Hamburg-Eppendorf, and DZHK (German Center for Cardiovascular Research), partner site Hamburg/Kiel/Lübeck, Hamburg, Germany. ^c^Faculty of Medicine and Health Sciences, Norwich Medical School, University of East Anglia NR4 7UQ, UK.

*Corresponding authors

^1^Equal contribution

Email: [j.bhagwan@axolbio.com](mailto:j.bhagwan@axolbio.com); [diogo.mosqueira@nottingham.ac.uk](mailto:diogo.mosqueira@nottingham.ac.uk); [chris.denning@nottingham.ac.uk](mailto:chris.denning@nottingham.ac.uk)

Tel: +44(0)1158231236

Fax: +44(0)1158231230

**Supplementary information contents:**

**Materials and Methods**

**5 Supplementary Figures + Legends**

**2 Supplementary Tables + Legends**

**1 Supplementary Video + Legend**

**References**

**Materials and Methods**

**2.1 hiPSC culture and cardiac differentiation**

hiPSCs were cultured and differentiated into cardiomyocytes following previously reported monolayer protocols [1], and quality control was performed by high-content imaging quantification of α-actinin positive cells, as previously described [2]. All patient skin biopsies were donated via informed consent under the approval of Research Ethics Committee - number 09/H0408/74). hiPSC-CM were dissociated by collagenase treatment as previously described [3] and replated in 5 µg/ml vitronectin-N (VN, Lifetech #A14700) coated vessels, and kept in RPMI+B27+insulin until phenotypic assays were performed.

**2.2 CRISPR/Cas9 genome editing to generate calcium reporter cell lines**

CRISPR/Cas9 genome editing and targeting was performed as described in the results section and [4]. Briefly, plasmid-based targeting was achieved by co-transfecting a targeting vector, gRNA(s) and Cas9 nuclease/Cas9 D10A nickase via Amaxa 4D nucleofection. Clones were then selected using puromycin, and genotyped by PCR screening and Sanger sequencing using primers from **Supplementary Table 1**. Clones with comparable RGECO expression between isogenic lines were selected for further phenotypic analysis, as previously described [5].

**2.3 Confocal analysis and ClampFit identification of abnormal calcium transients**

hiPSCs were differentiated in a monolayer protocol as previously described [1], and re-plated as single cells on day 15, at a density of 15,000 cells/cm^2^ in 5 µg/ml VN-coated 35 mm MatTek dishes, in serum-free RPMI+B27 without phenol red. 15 days later, intracellular calcium transient measurements were made in single cardiomyocytes using an LSM 880C confocal microscope (Carl Zeiss) in the line-scan mode as previously described [6]. Cardiomyocytes were located using a 40x oil objective and a longitudinal line was drawn across a single CM. The R-GECO1.0 fluorophore was excited with a 561 nm laser at 0.8% power, with a detection range of 579–639 nm. Line-scan images were taken every 75 milliseconds, with a pixel dwell time of 4.12 µsec, for a total of 4000 cycles resulting in a 5 minute scan. Cardiomyocytes were kept at 37°C and 5% CO_2_ throughout data acquisition. Independent experiments were performed in triplicate and at least 100 cardiomyocytes were analysed per replicate (single well) totalling 1,500-2,400 single cells for the *MYH7* isogenic set and 900-1,500 for the *ACTC1* duo.

Confocal line scan images were analysed in FiJi software (National Institutes of Health). The average fluorescence intensity of each line was calculated over time to give a confocal line-scan trace. Using the ‘multi kymograph’ function, a corresponding kymograph image was produced. In order to calculate beat rate and arrhythmic events, data were fed into pClamp software (Molecular Devices). Baselines were adjusted to account for photobleaching and calcium transients were counted and analysed using the ‘event detection’ function. Using the event viewer, any calcium transients that did not return to baseline and gave a ‘double peak’, or did not return to at least 75% of the previous calcium transient amplitude, were considered ‘abnormal’, as they reflect early or delayed afterdepolarisations that typically occur as a result of intracellular calcium overload [7].

**2.4 Optogenetics and 2D contractility measurements**

For optical pacing experiments, hiPSC-CMs were seeded at 115,000 cells/cm^2^ in a 96-well plate. An optogenetic CAG-ChR2-EYFP-T2A-BSD construct was transiently transfected into d26 hiPSC-CMs, no longer than 48h after dissociation, with ViaFect™ transfection reagent (Promega) in 20% FBS-enriched RPMI+B27 medium. Medium was replaced to serum-free RPMI+B27 24h later, and 72h after transfection optogenetic pacing experiments were carried out on the CellOPTIQ™ (Clyde Biosciences) platform at 37°C, 5% CO_2_. Using the OptoLED light source (Cairn Research), pulses of 480 nm blue light were applied at 1 Hz for 200 ms, with an average light intensity of 2 mW/mm^2^. Images were captured at 100 frames per second for a ten-second period using an ORCA-Flash4.0 digital CMOS camera (Hamamatsu: C11440). Contraction analysis was performed using a sum of absolute differences algorithm [8] within a proprietary ImageJ macro (Clyde BioSciences).

**2.5 Human Engineered Heart Tissue fabrication and 3D analysis of contractile force**

Human Engineered Heart Tissue (hEHTs) were fabricated as previously described [3] with each hEHT containing 1 million hiPSC-CMs freshly-dissociated after the monolayer differentiation protocol, embedded in fibrin and fed every other day for 2-3 weeks. Contractile force was analysed as previously described [9], whereby hEHTs were electrically paced (2 V, 2 Hz, impulse duration 4 ms) with carbon electrodes using a Grass S88X stimulator (Astro-Med). Automated video-optical recordings of silicone post deflection (EHT Technologies GmbH #C0001) and real-time calculation of contraction forces were performed with the EHT contraction analysis instrument (EHT Technologies GmbH #A0001) based on the known mechanical properties of the silicone posts. The contraction peaks were analysed in terms of frequency (beat rate), force, and contraction (T_1_) and relaxation time (T_2_) for 80% of peak height.

**2.6 SeaHorse analysis of metabolic activity**

The Seahorse XF96 extracellular flux analyser was used to assess mitochondrial respiration, as previously described [2], using the Mito Stress Kit (Agilent). Briefly, dissociated hiPSC-CM were seeded into VN-coated XF96 well plates, at a density of approximately 5,000 cells/mm^2^. Cardiomyocytes were cultured for one week as above and medium was exchanged for XF base medium (Agilent Technologies #102353), supplemented with 10 mM glucose (Sigma #G7528), 1 mM sodium pyruvate (Sigma #S8636) and 2 mM L-glutamine (Life Technologies #25030-081) 1h prior to the assay. The oxygen consumption rate (OCR) values were further normalized to the number of cells present in each well, quantified by 1:400 Hoechst33342 (Sigma # 14533) in PBS staining using fluorescence at 355 nm excitation and 460 nm emission in an automated imaging platform (CellaVista, Synentec).

**2.7 Gene expression analysis by qPCR**

Real-time qPCR reactions were performed via TaqMan^®^ Gene Expression Assays (Applied Biosystems) following manufacturer’s instructions. Briefly, Taqman^®^ mastermix (#4369016) including the probe of interest from **Supplementary Table 2** was added to a MicroAmp Fast 96 well plate (#4346907). Subsequently, cDNA samples (from initial 500 ng of reverse-transcribed RNA) from day 30 hiPSC-CMs were added to the plate and amplification was performed in ABI 7500 Real-Time PCR system. Normalisation was performed using the cardiac gene *TNNT2*. The average of WT isogenic cardiomyocytes lines was used for calculating relative quantification, using the ΔΔCT method [10].

**2.8 Immunocytochemistry and high-content imaging**

Immunocytochemistry and high-content imaging analysis were performed as previously described [2]. Dissociated hiPSC-CMs were cultured in VN-coated 96-well plates (CellCarrier, Perkin Elmer #6005550) at 100,000 cells/cm^2^ as described above. Cells were washed with PBS and fixed in 4% Paraformaldehyde (Sigma) at room temperature (RT) for 15 min. Afterwards, cells were washed in 0.1% Tween-20 (Fisher Scientific) in PBS, permeabilized with 0.1% Triton-X (Sigma) in PBS for 15 min at RT, and incubated with 4% goat serum (Sigma) in PBS (blocking solution) for 1h at RT, to prevent unspecific antibody binding. Subsequently, primary antibody incubation was performed overnight at 4°C in blocking solution, at the following dilutions: anti-RFP-1:1000 (Abcam: ab124754), anti-α-actinin-1:800 (Sigma: A7811), anti-NFAT2 1:1000 (Abcam: ab25916), anti-MEF2C 1:1000 (Abcam: ab227085). Thereafter, samples were washed 3 times with 0.1% Tween-20 in PBS and incubated with Alexa Fluor secondary antibodies (Life Technologies) in blocking solution for 1h at RT. Afterwards, cells were washed as above followed by nuclei counterstaining with 0.5 µg/ml DAPI (Sigma #D9542) in PBS for 30 min at RT. Samples were subsequently washed and stored at 4°C in PBS until automated image acquisition was performed in the Operetta high-content imaging system (Perkin Elmer) and analysed using Harmony high-content imaging analysis software, as previously described [2].

**2.9 Statistical analysis**

All data is presented as mean with standard deviation or box and whiskers plot, with the number of biological replicates indicated in the respective figure legend, performed in technical triplicates unless otherwise stated. Statistical analysis was performed using GraphPad software (v8.2). *ACTC1*^MUT/WT^ was directly compared to *ACTC1*^WT/WT^ hiPSC-CMs using unpaired Student’s t-test. A one-way ANOVA with Newman-Keuls multiple comparison test was used for *MYH7* isogenic trio data analysis, unless otherwise stated. Drug treatments were compared to vehicle controls using unpaired Student’s t-test. Significance tests were based on p-values as follows: * p<0.05; **p<0.01; ***p<0.001; ****p<0.0001.

**Supplementary Figures**

**Supplementary Figure 1 – CRISPR/Cas9 gene editing to generate isogenic HCM lines. A)** The g.*MYH7*^C9123T^ mutation was engineered in human induced pluripotent stem cells (hiPSCs) by dual gRNA/Cas9-nickase/CRISPR strategy followed by cassette excision. **B)** PCR genotyping of gene-edited clones confirms 1) recombination of homology sequence incorporating the single nucleotide polymorphism (SNP) of interest, 2) efficient removal of resistance cassette (correct product sizes indicated). **C)** Sanger sequencing validates the insertion of the g.MYH7-C9123T SNP in hiPSCs resulting in heterozygote and homozygote mutants. **D)** The c.*ACTC1*^G301A^ mutation was corrected from a heterozygous patient-derived hiPSC line using a gRNA/Cas9/CRISPR strategy followed by cassette excision. **E)** PCR genotyping demonstrates: 3) correct recombination of homology sequence in the host genome, 4) scarless excision upon recombination activity (correct product sizes indicated). **F)** Sanger sequencing confirms correction of heterozygote G/A variant into wild-type 301A genotype. LA - left arm of homology; RA - right arm of homology; TV - Targeting Vector; WT- wild-type; MUT- mutant; NTC - non-template control; MW - molecular weight.

**Supplementary Figure 2 – Performance of R-GECO indicator vs Fluo-4-AM dye for calcium imaging. A)** Fluorescence micrographs illustrating calcium signal of hiPSC-CMs treated with 10 μM Fluo-4-AM dye (left) vs stably expressing R-GECO (right) Scale bars = 50 μm. **B)** Percentage of cardiomyocytes expressing Fluo-4 AM or RGECO over time, relative to t=0 shows that Fluo-4-AM signal decreases more rapidly relative to R-GECO, where it is maintained even after 12h (N=4 biological replicates). **C)** Titration of CellTiter-Glo® luminescence kit for evaluating viability of hiPSC-CMs (N=3 biological replicates). **D)** Viability of hiPSC-CMs evaluated by this kit shows toxicity of Fluo-4-AM dye 1h post treatment, even at 100 nM (N=3 biological replicates). **E)** In contrast, R-GECO-expressing hiPSC-CMs show no toxicity relative to untargeted/ unlabelled cells (control), N=4-12 biological replicates. Data: mean±SD, unpaired t-test in B, one-way ANOVA test + Dunnett’s correction relative to no dye control in D and E.

**Supplementary Figure 3 – Properties of hiPSC-CM purity and intracellular calcium. A)** Fluorescent micrographs illustrating that RGECO-expressing calcium reporter lines showed similar cardiomyocyte differentiation efficiencies, evidenced by **B)** over 90% α-actinin positive cells, quantified by high-content imaging (N=10-20 independent biological replicates, scale bar = 50 μm). **C)** Representative calcium transient acquired by confocal line scanning of a single RGECO-expressing hiPSC-CM. Quantification of the baseline RGECO fluorescence intensity (F0) in cardiomyocytes showed that **D**) *MYH7*-mutant and **E**) *ACTC1*-mutant hiPSC-CMs displayed higher diastolic calcium levels relative to their respective healthy isogenic controls (~2.3-2.7-fold higher in *MYH7*-mutants, ~2.5-fold higher in *ACTC1*-mutants). **F)** Evaluation of SR Ca^2+^ release by optical mapping upon treatment with 10 mM caffeine showed no differences between isogenic lines (N=3 independent biological replicates). **G)** Representative confocal line scans of isogenic *MYH7* hiPSCs and **H)** *ACTC1*-hiPSCs upon treatment with caffeine (at t=30s, highlighted by dotted line). Data: mean±SD, one-way ANOVA + Newman-Keuls correction for multiple comparisons in *MYH7* isogenic trio and unpaired t-test between *ACTC1* lines, *p<0.05.

**Supplementary Figure 4 – Transfection of hiPSC-CMs to enable optogenetic pacing. A)** A vector containing a channelrhodopsin-2 (ChR2) protein driven by CAG promoter and eYFP tagging was generated. **B)** A reporter eGFP plasmid was used to test Viafect™ transfection reagent in cardiomyocytes. **C)** eYFP expression identified ChR2^+^ hiPSC-CMs, amenable to optical pacing (Scale bar = 50 µm). **D)** Transfection efficiency of eGFP plasmid was ~56% while about ~38% of cardiomyocytes expressed ChR2 (N=6-37 biological replicates). **E)** hEHTs showed differences in spontaneous beat rates between *MYH7* isogenic set (~106±4.2 bpm in WT/WT vs ~127±9.6 bpm in WT/MUT and 120±7.3 bpm in MUT/MUT, N=4-8 independent biological replicates), but not in the ACTC1 duo (~118±3.5 bpm in WT/WT vs 115±2.4 bpm in WT/MUT, N=3-11 independent biological replicates). Data: mean±SD, one-way ANOVA + Newman-Keuls correction for multiple comparisons in *MYH7* isogenic trio and unpaired t-test between *ACTC1* lines, **p<0.01, ***p<0.001.

**Supplementary Figure 5 – Pharmacological intervention approaches attempting to rescue HCM phenotypes.** Quantification of frequency of arrhythmic events measured by confocal line scanning in **A)** *MYH7* isogenic trio and **B)** *ACTC1* set of hiPSC-CMs treated with 10 µM dantrolene shows modest amelioration of arrythmogenic phenotype relative to their respective vehicle controls, 0.1% v/v DMSO (~13-48% reduction in *MYH7*-mutant lines, N=4-13 biological replicates; ~38% decrease in *ACTC1* heterozygous mutants, N=4-7 biological replicates). Representative traces of optically-paced (1 Hz) ChR2^+^ g.*MYH7*^C9123T^ hiPSC-CMs treated with **C,F)** 0.1% (v/v) DMSO (vehicle control), **D,H)** 0.1 µM Omecamtiv mecarbil (OM) and **E,H)** 1 µM OM, relative to **I)** WT isogenic control. **J)** Quantification of contractility in 2D CellOPTIQ™ shows that treatment with OM did not restore hypo-contractile phenotype in *MYH7*-mutant hiPSC-CMs back to baseline healthy levels (N=3 biological replicates). Data: mean ±SD or box and whiskers plots, unpaired t-test between vehicle control and drug-treated condition per hiPSC-CM line (*p<0.05).

**Supplementary Table 1 – Primers used for PCR genotyping and Sanger sequencing genome-edited hiPSCs**

**Supplementary Table 2 - Taqman^®^ (Applied Biosystems) probes used for qRT-PCR.**

**Supplementary Video 1 – Confocal line scanning showing spontaneously beating hiPSC-CMs expressing RGECO1.0.**

**References**

[1] D. Mosqueira, I. Mannhardt, J.R. Bhagwan, K. Lis-Slimak, P. Katili, E. Scott, M. Hassan, M. Prondzynski, S.C. Harmer, A. Tinker, J.G.W. Smith, L. Carrier, P.M. Williams, D. Gaffney, T. Eschenhagen, A. Hansen, C. Denning, CRISPR/Cas9 editing in human pluripotent stem cell-cardiomyocytes highlights arrhythmias, hypocontractility, and energy depletion as potential therapeutic targets for hypertrophic cardiomyopathy, Eur. Heart J. 39(43) (2018) 3879-3892.

[2] D. Mosqueira, K. Lis-Slimak, C. Denning, High-Throughput Phenotyping Toolkit for Characterizing Cellular Models of Hypertrophic Cardiomyopathy In Vitro, Methods Protoc. 2(4) (2019).

[3] K. Breckwoldt, D. Letuffe-Breniere, I. Mannhardt, T. Schulze, B. Ulmer, T. Werner, A. Benzin, B. Klampe, M.C. Reinsch, S. Laufer, A. Shibamiya, M. Prondzynski, G. Mearini, D. Schade, S. Fuchs, C. Neuber, E. Kramer, U. Saleem, M.L. Schulze, M.L. Rodriguez, T. Eschenhagen, A. Hansen, Differentiation of cardiomyocytes and generation of human engineered heart tissue, Nat. Protocols 12(6) (2017) 1177-1197.

[4] A. Kondrashov, M. Duc Hoang, J.G.W. Smith, J.R. Bhagwan, G. Duncan, D. Mosqueira, M.B. Munoz, N.T.N. Vo, C. Denning, Simplified Footprint-Free Cas9/CRISPR Editing of Cardiac-Associated Genes in Human Pluripotent Stem Cells, Stem Cells Dev. 27(6) (2018) 391-404.

[5] J. Bhagwan, E. Collins, D. Mosqueira, M. Bakar, B. Johnson, A. Thompson, J. Smith, C. Denning, Variable expression and silencing of CRISPR-Cas9 targeted transgenes identifies the AAVS1 locus as not an entirely safe harbour, F1000Research 8(1911) (2019).

[6] M. Yazawa, B. Hsueh, X. Jia, A.M. Pasca, J.A. Bernstein, J. Hallmayer, R.E. Dolmetsch, Using induced pluripotent stem cells to investigate cardiac phenotypes in Timothy syndrome, Nature 471(7337) (2011) 230-4.

[7] S. Wagner, S. Maier Lars, M. Bers Donald, Role of Sodium and Calcium Dysregulation in Tachyarrhythmias in Sudden Cardiac Death, Circ. Res. 116(12) (2015) 1956-1970.

[8] T. de Korte, P. Katili, N. Mohd Yusof, B. van Meer, U. Saleem, F. Burton, G. Smith, P. Clements, C. Mummery, T. Eschenhagen, A. Hansen, C. Denning, Unlocking Personalized Biomedicine and Drug Discovery with Human Induced Pluripotent Stem Cell–Derived Cardiomyocytes: Fit for Purpose or Forever Elusive?, Annu. Rev. Pharmacol. Toxicol. 60 (2020) 529-551.

[9] I. Mannhardt, K. Breckwoldt, D. Letuffe-Brenière, S. Schaaf, H. Schulz, C. Neuber, A. Benzin, T. Werner, A. Eder, T. Schulze, B. Klampe, T. Christ, Marc N. Hirt, N. Huebner, A. Moretti, T. Eschenhagen, A. Hansen, Human Engineered Heart Tissue: Analysis of Contractile Force, Stem Cell Reports 7(1) (2016) 29-42.

[10] T.D. Schmittgen, K.J. Livak, Analyzing real-time PCR data by the comparative CT method, Nat. Protocols 3(6) (2008) 1101-1108.
